# Greater amount of lying and reclining associate with cardiovascular disease risk score and several risk factors, while short sitting bouts and standing have opposite relation

**DOI:** 10.1016/j.ajpc.2025.101327

**Published:** 2025-10-09

**Authors:** Pauliina Husu, Henri Vähä-Ypyä, Kari Tokola, Harri Sievänen, Onni Niemelä, Tommi Vasankari

**Affiliations:** a The UKK Institute for Health Promotion Research, Kaupinpuistonkatu 1, 33500, Tampere, Finland; b Department of Laboratory Medicine and Medical Research Unit, Seinäjoki Central Hospital and University of Tampere, Kalevantie 4, Tampere, FI-33014, Finland; c Faculty of Medicine and Health Technology, University of Tampere, Kalevantie 4, Tampere, FI-33014, Finland

**Keywords:** Cardiometabolic health, Cardiovascular risk factors, Sedentary behavior, Stationary behavior, Physical Activity

## Abstract

Excess sedentary behavior (SB) seems to be harmful for health, whereas the effects of standing can be opposite. The present study aimed at 1) describing different components of SB (lying, reclining, sitting) and standing accumulating from different bout lengths in a population-based sample and 2) analyzing their associations with indicators of cardiometabolic health. The study is based on cross-sectional accelerometer-measured data on 24/7 physical behavior among 20–69-year-old Finns. Outcomes were Framingham score for cardiovascular disease (CVD) risk, serum high (HDL)- and low-density lipoprotein (LDL) and total cholesterol, triglycerides, and waist circumference. Participants (*n* = 4298) mean age was 51 years (SD=13) and 61 % were female. More lying and reclining, regardless of bout length, were associated with higher CVD-score (*p* ≤ 0.001), lower HDL-cholesterol (*p* < 0.001), higher triglycerides (*p* < 0.001) and larger waist circumference (*p* < 0.001). Longer sitting time accumulating from <30 min bouts was associated with lower CVD-score (*p* < 0.001), higher HDL- (*p* < 0.001), lower LDL- (*p* = 0.004) and total cholesterol (*p* = 0.009), lower triglycerides (*p* < 0.001) and smaller waist circumference (*p* < 0.001). Longer sitting accumulating from bouts exceeding 20 min was associated with larger waist circumference (*p* < 0.001) indicating that health associations of sitting may depend on bout length. More standing regardless of bout length was associated with lower CVD-score (≤0.001), higher HDL-cholesterol (*p* < 0.001), lower triglycerides (*p* < 0.001) and smaller waist circumference (*p* < 0.001). These associations were mostly independent of moderate-to-vigorous physical activity. Lying and reclining had negative associations with CVD-score and risk factors while short sitting bouts and standing had positive associations, underpinning the importance of evaluating the different components of stationary behavior separately without combining them to overall SB.

## 1 Introduction

Sedentary behavior (SB) is one of the key components of the human movement spectrum [1] conferring a significant impact on health and wellbeing [[2], [3], [4]] and covering an increasing proportion of waking hours [[5], [6], [7]]. SB refers to any waking behavior expending ≤1.5 metabolic equivalents (METs), while being in a sitting, reclining, or lying posture [8]. Common sedentary behaviors include TV viewing, video game playing, screentime, sitting in automobiles, and reading [9]. If standing position without ambulatory movement is included, the term stationary behavior should be used instead of SB [8].

Excessive and prolonged SB is harmful for health [10]. It can lead to metabolic problems like insulin resistance, vascular dysfunction, deposition of visceral fat, deviations in blood lipid, and low-grade inflammation as well as to decreased functional ability due to reduced cardiorespiratory fitness, loss of muscle mass and strength, bone loss, and increased total body fat mass [4]. Especially prolonged sitting over 8 h induces deterioration of arterial blood flow, shear rate, and microcirculation in lower limb muscles [11]. Further SB is associated with all-cause mortality, partly independently of physical activity (PA) [12,13]. Accumulating >22 min of moderate to vigorous PA (MVPA) per day seems to eliminate the increased mortality risk of high sedentary time [13], and MVPA also has potential to eliminate other health risks associated with excess SB [4,11] (e.g. insulin resistance, vascular dysfunction, deposition of visceral fat, low-grade inflammation, decreased functional ability, loss of muscle mass and strength, bone loss, and increased total body fat mass). Based on a recent review high SB time, especially when combined with low PA, seems to be associated with poor cardiopulmonary fitness and worse long-term prognosis among patients with acute coronary syndrome [14]. Thus, it is important to consider PA levels when assessing the health associations of SB.

Besides the total time of sedentary or stationary behavior, there is a need to identify different body postures within it [15]. Stationary body postures differ from each other in terms of metabolic, cardiovascular, and muscular functions. Sitting is associated with higher energy expenditure, heart rate, skeletal muscle blood flow, and contractive activity than reclining [4]. Compared to sitting, standing is associated with higher muscle activity, fat oxidation, and circulating glucose level [16], but lower than those of PA performed at any intensity [4]. Further, higher SB is associated with metabolic inflexibility while standing and PA of any intensity is associated with metabolic flexibility [17]. Different body postures (lying, reclining, sitting, and standing) relate differently also with body mass index and cardiorespiratory fitness [15]. However, health effects of standing have been studied much less than those of total SB. Studying both sitting and standing Ahmadi et al. [18] reported that standing time was not associated with cardiovascular disease (CVD) risk but was associated with higher orthostatic circulatory disease risk. On the other hand sitting time exceeding 10 h/day was associated with both higher orthostatic circulatory disease and major CVD risk [19]. Thus, the so far published deleterious associations of overall stationary time are primarily driven by sitting.

Besides total times, it is important to consider specifically how sedentary or stationary time is accumulated [10,[20], [21], [22]]. Young adults seem to be more sedentary than middle-aged adults, but they spend their SB in shorter, more frequent bouts [21]. The number of SB periods, especially the ones lasting longer than 10 min at the time, seem to be more strongly associated with the CVD risk than the total sedentary time [22]. Frequently interrupted sitting has beneficial associations with several cardio-metabolic biomarkers, while prolonged periods of sitting may exacerbate harmful effects [23]. Short SB bouts seem to have favorable and long bouts unfavorable associations at least with adiposity markers [24]. Further, greater daily numbers of sit-to-stand transitions and <5 min standing bouts seem to be associated with a smaller waist circumference even when cardiorespiratory fitness and MVPA are accounted for [25]. Besides body composition, both the reduction of prolonged SB bouts and frequent interruptions of SB have led to benefits in fasting glucose, insulin, HbA1c, HDL-concentrations, systolic blood pressure, vascular function [4], as well as in HOMA-insulin resistance, triglycerides, liver enzymes, and resting heart rate [26]. In addition, reduced SB seems to improve tissue-specific insulin sensitivity in postural muscles [27]. Thus, reduced SB can lessen many cardiometabolic risks, but more intense PA than just replacing sitting time by standing may be required [28].

In many studies the term SB is used to refer to all activities with no ambulatory movement [10,17,20,24]. Due to different physiological effects of the SB components [4], it can be expected that also the health associations differ from each other. Many population-based studies have not measured or analyzed standing as a distinct behavior, but often it has been regarded either as a part of SB or light PA [10,24,29]. Further, many previous studies have analyzed only the total SB time without considering its accumulation from different bout lengths [13,30]. Thus, the objective of the present study was to describe the components of stationary behavior in population-based samples of working-aged adults and to analyze their associations with CVD risk score and selected cardiometabolic risk factors considering also different bout lengths and MVPA level. We hypothesized that the direction of associations would differ between the components of stationary behavior, and that longer bout lengths would have more negative associations than the shorter ones.

## 2 Methods

This study is based on two cross-sectional population-based studies, FINFIT2017 and FINFIT2021, which are multifactorial studies on PA, fitness, and health conducted with stratified random samples of 20–69-year-old Finnish adults [6,31]. Potential participants were drawn from the population registry of seven city-centered regions in Finland: Helsinki, Turku, Tampere, Kuopio, Jyväskylä, Oulu, and Rovaniemi regions spread across both sexes in five age groups (20–29, 30–39, 40–49, 50–59, and 60–69 years). Other inclusion or exclusion criteria were not used. Invitation letters containing information about the study and informed consent with the option to withdraw from the study at any time were mailed to 13,500 potential participants in FINFIT2017 and to 16,500 in FINFIT2021. The recruitment periods were from August 2017 to March 2019, and from August 2021 to April 2022. The data collection comprised three parts: (1) health and fitness examinations, including measurements of waist circumference, height and weight, cardiorespiratory and musculoskeletal fitness and fasting blood samples, at the local research centers; (2) accelerometer-measured PA, standing, SB, and time in bed (TIB) 24/7 over one week (7 consecutive days); and (3) a health-related questionnaire either online or in paper form [31].

The present study included 4298 participants who wore accelerometers for at least four days 24 h per day. The studies were carried out in accordance with the Declaration of Helsinki and the Regional Ethics Committee of the Expert Responsibility Area of Tampere University Hospital approved them (R17030 and R2105). All participants gave signed informed consent before participation.

### 2.1 Variables

#### 2.1.1 Metabolic health indicators

Indicators of metabolic health included Framingham risk score (FRS) for CVD risk [32], and the risk factors: serum high- and low-density lipoprotein (HDL and LDL), and total cholesterol, triglyceride, and measured waist circumference. The FRS was calculated based on age, serum total and HDL-cholesterol, systolic blood pressure, self-reported smoking, and diabetes status [32]. Instead of antihypertensive medication used by D’Agostino et al. [32] to calculate FRS we used self-reported hypertension to calculate the score [22]. Besides FRS the CVD risk was assessed also in terms of Systematic Coronary Risk Evaluation model (SCORE2) [33] based on sex, age, serum total and HDL-cholesterol, systolic blood pressure, self-reported smoking, and moderate risk region. These two scores had corresponding associations with stationary behavior (supplementary Tables 1A-C), and the FRS was chosen as the main outcome since our previous study showed PA- and SB-related validity for it [22].

Fasting blood samples were taken after a 12 h overnight fasting, and data for waist circumference and blood pressure were measured at the health and fitness examinations conducted at the local research centers. Blood pressure was measured on the non-dominant arm of the seated participants, and the mean of two measurements was used. Waist circumference was measured as a part of a standard pre-testing health screening by educated fitness testers [31].

Serum and plasma were separated by centrifugation (2000 g for 10 min) and stored at −75 °C prior to analysis. Concentrations of serum total cholesterol, HDL- and LDL-cholesterol, and triglyceride were analyzed using standard enzymatic methods on a Cobas c702 clinical chemistry analyzer (Roche Diagnostics). All measurements were carried out in an SFS-EN ISO 15189:2013 accredited laboratory blind to the knowledge of the demographic and physical activity characteristics of the participants. Data for hypertension, smoking and diabetes status were self-reported by a study questionnaire.

#### 2.1.2 Measurement of physical behavior

Participants’ physical behavior in terms of time in bed (TIB), SB (lying, reclining, sitting), standing, light PA, moderate PA, and vigorous PA was measured by a tri-axial accelerometer (UKK RM42, UKK Terveyspalvelut Oy, Tampere, Finland) 24/7. During waking hours, the accelerometer was attached to an elastic belt and worn on the right side of hip, excluding water-based activities. For the assessment of TIB, the accelerometer was moved from the belt to an adjustable wristband and attached to the non-dominant wrist. The accelerometer collected and stored the raw triaxial data in actual g-units in ±16 G range at a 100 Hz sampling rate [31].

During waking hours, the mean amplitude deviation (MAD) was calculated from the resultant acceleration signal in 6 s epochs and the epoch-wise acceleration values were converted to METs [33]. The epoch-wise MET values were further smoothed by calculating a one-minute exponential moving average. Using the smoothed MET values, the total PA was classified as light PA (1.5–2.9 METs), moderate PA (3.0–5.9 METs), or vigorous PA (6 METs or more). In the present study, moderate and vigorous PA were combined into MVPA and analyzed in age groups- and sex-specific thirds (low, middle and high).

Since the present study focused on stationary behavior during waking hours, the epochs with <1.5 METs (i.e. MAD value <22.5 mg) were further analyzed with the angle for posture estimation (APE) method [15,34]. The APE method is based on two concepts: the Earth’s gravity vector is constant, and the body posture during walking is upright. Accelerometer orientation relative to the gravity vector is calculated as the epoch-wise mean acceleration across the three (x, y and z) axes. The accelerometer orientation during walking is taken as the reference vector. The APE denotes the angle between the measured epoch vector and reference vector. The accelerometer orientation during walking was used as a reference value and the recognition of walking was based on the intensity of activity, step rate and movement steadiness. The APE method is validated both under laboratory conditions through direct posture observation by researchers, and in real-life settings against a thigh-worn accelerometer [15,34]. In real-life settings, the APE method has shown over 90 % accuracy in classifying body postures [34].

APE values <11.6° denoted standing, those between 11.6° and 30° sitting, the values between 30° and 73.9° reclining, and values exceeding 73.9° lying [15]. Besides the total times, different bout lengths (<3 min, <5 min, <10 min, <20 min, <30 min, <60 min, ≥1 min, ≥3 min, ≥5 min, ≥10 min, ≥20 min, ≥30 min, and ≥60 min) and ranges (<10 min, 10–19.9 min, 20–29.9 min, 30–59.9 min and ≥60 min) of the stationary behavior components were analyzed. Analyses of the associations between components of stationary behavior and indicators of metabolic health were conducted also by taking the mean daily MVPA time in thirds into account.

### 2.2 Background characteristics

Marital status, educational level, and body mass index (BMI) were used as background factors. BMI was calculated based on weight and height measured at the health and fitness examinations: body mass divided by height squared (kg/m^2^). Marital status and educational level were assessed by the study questionnaire. Marital status was assessed by asking the participants to report whether they were single, cohabited, married, widowed, or divorced [31]. Education was assessed by asking the participants to report their highest education level completed after primary school. The responses were categorized into four groups: (1) no vocational education, (2) vocational education, (3) bachelor’s degree, (4) master’s degree or higher.

### 2.3 Statistical analysis

GLM (multivariate regression) adjusted for age group and sex was used to test the associations between components of stationary behavior and cardiometabolic risk factors. The associations were also analyzed separately in the MVPA thirds (low, middle and high) with standardized beta coefficients (Beta). Both standardized and non-standardized beta coefficients (B) were used to analyze the strength and direction of associations between the stationary behavior bouts and indicators of metabolic health. Standardized beta coefficients are presented in Fig. 3 and non-standardized coefficients (B) in supplementary Table 3. All analyses were conducted using the SPSS (IBM SPSS Statistics, V.29).

## 3 Results

Majority (61 %) of the 4298 participants were females. The participants’ mean age was 50.4 years (SD 13.2). More than half of the participants were married, one fourth had master’s degree. Less than half of the participants were normal weight in terms of BMI less than 25 kg/m^2^ (Table 1). Slightly over one tenth of the participants were smokers, males more than females. More males than females reported also hypertension. Less than one tenth of participants reported to have diabetes. Cholesterol levels and waist circumference of the participants were slightly over the target ranges both in males and females.

**Table 1:** The background characteristics of the participants and p-values for sex difference.

		Men	Women	p-value*	Total
Age group ( %)	20–29 years	5.9	8.9	<0.001	7.7
	30–39 years	14.5	16.3		15.6
	40–49 years	19.1	21.6		20.7
	50–59 years	24.4	24.2		24.3
	60–69 years	36.1	28.9		31.7
Marital status ( %)	single	11.1	14.8	<0.001	13.4
	cohabited	20.9	20.1		20.4
	married	60.6	51.2		54.7
	widowed/divorced	7.3	13.9		11.4
Education ( %)	no vocational education	11.5	7.4	<0.001	8.9
	vocational education	45.8	43.9		44.6
	Bachelor's degree	18.8	22.9		21.3
	Master's degree	23.9	25.8		25.1
Body mass index ( %)	≤24.9	32.5	49.3	<0.001	42.5
	25.0–29.9	48.3	32.4		38.8
	≥30.0	19.1	18.4		18.7
Current smoker ( %)	Yes	15.0	8.2	<0.001	10.8
	No	85.0	91.2		89.2
Diabetes ( %)	Yes	8.2	5.9	0.007	6.8
	No	91.8	94.1		93.2
Hypertension	Yes	24.9	17.8	<0.001	20.5
( %)	No	75.1	82.2		79.5
CVD risk score (FRS)	Mean (SD)	0.14 (0.09)	0.07 (0.06)	<0.001	0.10 (0.08)
HDL cholesterol, mmol/l	Mean (SD)	1.5 (0.4)	1.8 (0.4)	<0.001	1.7 (0.5)
LDL cholesterol, mmol/l	Mean (SD)	3.2 (0.9)	3.1 (0.9)	<0.001	3.1 (0.9)
Total cholesterol, mmol/l	Mean (SD)	5.0 (1.0)	5.1 (1.0)	0.012	5.1 (1.0)
Triglyceride, mmol/l	Mean (SD)	1.3 (0.8)	1.1 (0.5)	<0.001	1.2 (0.6)
Waist circumference, cm	Mean (SD)	97.2 (12.2)	87.9 (13.4)	<0.001	91.6 (13.7)

*2-sided T-test for the continuous variables, and Chi2 -test for the categorical variables.

CVD=cardiovascular disease, HDL=high-density lipoprotein, LDL=low-density lipoprotein.

**Fig. 1:**
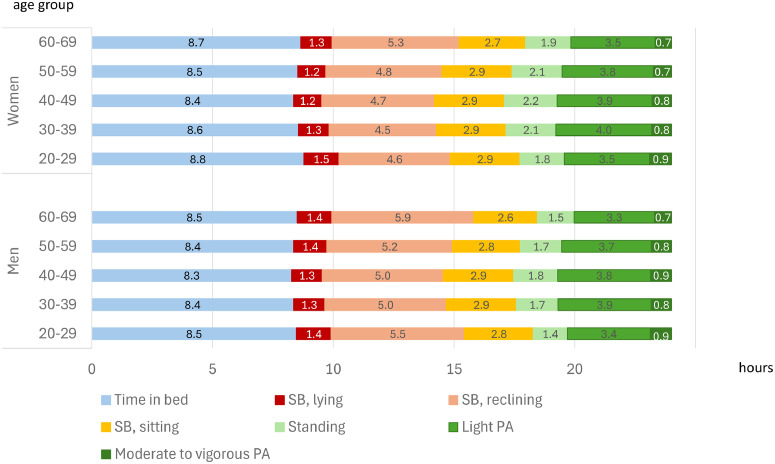
Different intensities of physical activity, different modes of stationary behavior and time in bed among the participants.

Fig. 1 describes the participants’ physical behavior in age groups and by sex. Participants spent on average 8 h 30 min per day in TIB. Females had on average 9 min longer TIB than males (*p* < 0.001). On average nine hours 10 min per day was spent sedentary, of which 1 h 18 min was spent lying, 5 h 3 min in reclining and 2 h 50 min in sitting posture. Standing covered on average 1 h 52 min. Males had on average 5.5 (95 % CI: 1.9 to 9.0) minutes more lying and 34.4 (28.1 to 40.7) minutes more reclining during waking hours than females, while standing was more prevalent among females (*p* < 0.001). Both males and females in the oldest age group had on average more reclining than the younger age groups (*p* < 0.05). Among females, the youngest age groups spent more time lying during waking hours than the older groups (*p* < 0.01). Majority of the PA comprised of light intensity activities. MVPA covered less than an hour per day. Females had on average more light PA than males (*p* < 0.001), while males had on average slightly more MVPA than females (*p* = 0.025). The older age groups were slightly less active than the younger ones (*p* < 0.01).

**Fig. 2:**
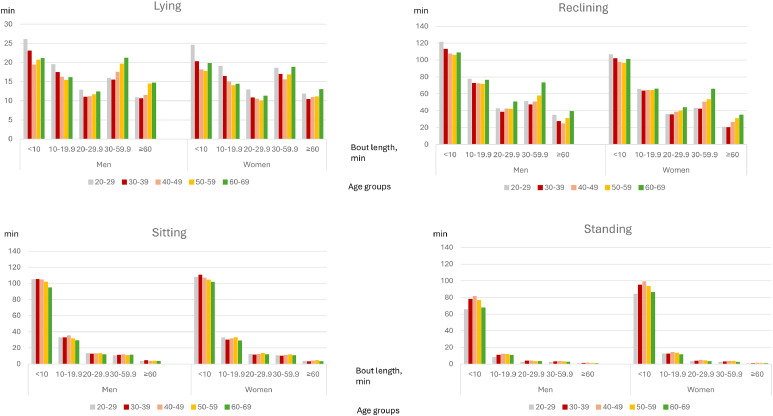
Stationary behavior accumulating from different bout lengths.

Lying during waking hours accumulated mostly from short, <10 min bouts and from the bouts exceeding 30 min (Fig. 2, Supplementary Table 2). In males, the youngest age group accumulated on average more lying from the short bouts than the participants aged over 40 years (*p* < 0.01). In females, the youngest age group differed from all the other age groups regarding <10 min (*p* < 0.001), 10–19.9 min (*p* < 0.01) and 20–29.9 min (*p* < 0.05) bouts. Regarding the bouts exceeding 30 min, only the oldest age group differed from the age groups younger than 50 years, especially among the males (*p* < 0.05) (Supplementary Table 2).

Reclining accumulated mostly from <10 min bouts.The youngest participants (20–29 years) accumulated on average more reclining from these short bouts than the older groups (*p* < 0.05). The oldest participants (60–69 years) accumulated more reclining from longer, 20–29.9 min, 30–59.9 min and at least 60 min (*p* < 0.001) bouts. Also sitting and standing accumulated mostly from short, <10 min, bouts. The oldest participants accumulated on average less sitting from these short bouts than the younger age groups (*p* < 0.05). Regarding standing, the youngest (20–29 years) and oldest (60–69 years) participants accumulated on average less time from short bouts than the participants aged 30–59 years (*p* < 0.05).

All components of stationary behavior during waking hours were associated with CVD risk score (FRS) and waist circumference. As to the strength and direction of the associations, more total time spent in lying or reclining were associated with higher FRS, higher triglyceride levels, and larger waist circumference (positive standardized beta coefficient) (Table 2), while more sitting and standing were associated with lower FRS, lower level of triglycerides, and narrower waist (negative standardized beta coefficient). These associations were statistically significant in all MVPA groups.

**Table 2:** Associations between components of stationary behavior and indicators of metabolic health taking moderate-to-vigorous physical activity into account. GLM adjusted for age group and sex.

			Beta*	95 % CI	p-value
CVD risk score (FRS)	SB, lying	low MVPA	149.4	84.6;214.3	<0.001
		middle MVPA	137.5	76.3;198.6	<0.001
		high MVPA	137.9	79.7;196.0	<0.001
		total	150.3	109.8;190.9	<0.001
	SB, reclining	low MVPA	251.8	139.7;363.4	<0.001
		middle MVPA	197.3	86.2;308.5	0.001
		high MVPA	218.0	113.1;322.9	<0.001
		total	191.6	118.2;265.0	<0.001
	SB, sitting	low MVPA	−173.0	−252.7;−93.3	<0.001
		middle MVPA	−202.4	−282.5;−122.4	<0.001
		high MVPA	−182.6	−260.9;−104.4	<0.001
		total	−146.5	−200.5;−92.4	<0.001
	Standing	low MVPA	−179.0	−250.1;−107.8	<0.001
		middle MVPA	−179.4	−251.9;−107.7	<0.001
		high MVPA	−153.1	−223.4;−82.8	<0.001
		total	−153.2	−200.2;−106.3	<0.001
HDL-cholesterol	SB, lying	low MVPA	−17.0	−25.3;−8.8	<0.001
		middle MVPA	−9.1	−16.3;−1.9	<0.001
		high MVPA	−2.3	−8.7;4.0	0.473
		total	−8.9	−13.2;−4.6	<0.001
	SB, reclining	low MVPA	−31.8	−45.8;−17.7	<0.001
		middle MVPA	−26.6	−39.4;−13.7	<0.001
		high MVPA	−25.6	−37.0;−14.2	<0.001
		total	−35.3	−42.8;−27.8	<0.001
	SB, sitting	low MVPA	18.6	8.5;28.7	<0.001
		middle MVPA	12.8	3.5;22.0	0.007
		high MVPA	5.4	−3.3;13.9	0.223
		total	5.3	−0.3;10.9	0.063
	Standing	low MVPA	24.0	15.3;32.9	<0.001
		middle MVPA	28.9	20.9;36.9	<0.001
		high MVPA	17.9	10.6;25.3	<0.001
		total	23.4	18.8;28.0	<0.001
LDL-cholesterol	SB, lying	low MVPA	1.5	−2.1;5.0	0.412
		middle MVPA	1.1	−2.2;4.5	0.493
		high MVPA	1.9	−1.4;5.2	0.260
		total	0.4	−1.7;2.4	0.745
	SB, reclining	low MVPA	−0.6	−6.7;5.5	0.844
		middle MVPA	3.2	−2.8;9.3	0.291
		high MVPA	3.2	−2.7;9.2	0.286
		total	4.6	1.0;8.3	0.013
	SB, sitting	low MVPA	−4.6	−8.9;−0.2	0.039
		middle MVPA	−2.2	−6.5;2.1	0.309
		high MVPA	−4.4	−8.8;0.1	0.054
		total	−4.4	−7.1;−1.7	0.001
	Standing	low MVPA	−2.5	−6.4;1.3	0.190
		middle MVPA	−0.5	−4.3;3.2	0.779
		high MVPA	−3.2	−7.1;0.6	0.098
		total	−3.3	−5.5;−1.0	0.004
Total cholesterol	SB, lying	low MVPA	1.0	−2.3;4.3	0.553
		middle MVPA	0.7	−2.4;3.8	0.663
		high MVPA	2.0	−1.0;5.0	0.193
		total	0.1	−1.8;2.1	0.887
	SB, reclining	low MVPA	−2.7	−8.3;2.9	0.347
		middle MVPA	1.6	−3.9;7.2	0.566
		high MVPA	−0.8	−6.3;4.6	0.768
		total	1.4	−2.1;4.7	0.437
	SB, sitting	low MVPA	−3.2	−7.2;0.9	0.124
		middle MVPA	−1.6	−5.6;2.4	0.438
		high MVPA	−4.0	−8.1;0.1	0.055
		total	−4.0	−6.5;−1.5	0.002
	Standing	low MVPA	−2.2	−5.7;1.3	0.221
		middle MVPA	0.4	−3.1;3.9	0.810
		high MVPA	−1.7	−5.2;1.9	0.352
		total	−2.2	−4.3;−0.2	0.036
Triglyceride	SB, lying	low MVPA	12.2	7.8;16.7	<0.001
		middle MVPA	9.8	5.0;14.6	<0.001
		high MVPA	9.1	3.8;14.5	0.001
		total	11.5	8.8;14.2	<0.001
	SB, reclining	low MVPA	13.0	5.3;20.8	0.001
		middle MVPA	23.3	14.8;31.8	<0.001
		high MVPA	18.7	9.1;28.4	<0.001
		total	22.0	17.1;26.9	<0.001
	SB, sitting	low MVPA	−10.6	−16.2;−5.1	<0.001
		middle MVPA	−10.9	−17.0;−4.7	0.001
		high MVPA	−10.2	−17.5;−3.0	0.006
		total	−8.8	−12.3;−5.3	<0.001
	Standing	low MVPA	−16.1	−20.9;−11.3	<0.001
		middle MVPA	−16.8	−22.1;−11.5	<0.001
		high MVPA	−15.1	−21.3;−8.9	<0.001
		total	−15.9	−19.0;−12.9	<0.001
Waist circumference	SB, lying	low MVPA	0.4	0.2;0.6	<0.001
		middle MVPA	0.6	0.3;0.8	<0.001
		high MVPA	0.3	0.1;0.6	0.007
		total	0.5	0.3;0.6	<0.001
	SB, reclining	low MVPA	2.1	1.8;2.5	<0.001
		middle MVPA	2.0	1.6,2.4	<0.001
		high MVPA	1.5	1.1;1.9	<0.001
		total	2.0	1.7;2.2	<0.001
	SB, sitting	low MVPA	−0.9	−1.1;−0.6	<0.001
		middle MVPA	−0.7	−1.0;−0.4	<0.001
		high MVPA	−0.4	−0.7;−0.1	0.022
		total	−0.7	−0.8;−0.5	<0.001
	Standing	low MVPA	−1.4	−1.6;−1.2	<0.001
		middle MVPA	−1.6	−1.9;−1.4	<0.001
		high MVPA	−1.2	−1.5;−1.0	<0.001
		total	−1.2	−1.3;−1.1	<0.001

* standardized beta coefficients CVD=cardiovascular disease, FRS=Framingham risk score, HDL=high-density lipoprotein, LDL=low-density lipoprotein, SB=sedentary behavior, MVPA=moderate-to-vigorous physical activity, CI=Confidence interval.

More lying and reclining were associated also with lower HDL-cholesterol, while more standing was associated with higher HDL-cholesterol. The associations of reclining and standing were statistically significant in all MVPA levels. More lying was associated with lower HDL-cholesterol and more sitting with higher HDL-cholesterol only among those with low or middle level of MVPA.

Regarding LDL-cholesterol, the associations were opposite to those of HDL: more total time of reclining was associated with higher LDL-cholesterol while more total sitting and standing time were associated with lower LDL. Regarding the MVPA levels, the only statistically significant association was found among the participants with low MVPA, among whom a longer sitting time was associated with lower LDL level. Longer total sitting and standing times were associated with lower total cholesterol levels, but the associations were not statistically significant in any of the MVPA levels.

Regarding TIB the associations were corresponding to those of lying and reclining during waking hours. Longer total TIB was associated with higher CVD risk score (FRS), lower HDL-cholesterol, higher LDL-cholesterol, higher triglyceride and larger waist circumference (Table 3). Additionally, higher total TIB was associated with higher total cholesterol.

**Table 3:** Associations between total time in bed and indicators of metabolic health. GLM adjusted for age group and sex.

		Beta*	95 % CI	p-value
Time in bed	CVD risk score (FRS)	0.13	0.06;0.21	0.001
	HDL-cholesterol	−0.09	−0.15;−0.04	<0.001
	LDL-cholesterol	0.08	0.03;0.13	0.002
	Total cholesterol	0.09	0.04;0.14	0.001
	Triglyceride	0.10	0.05;0.15	<0.001
	Waist circumference	0.09	0.03;0.14	0.002

* standardized beta coefficients CVD=cardiovascular disease, FRS=Framingham risk score, HDL=high-density lipoprotein, LDL=low-density lipoprotein, CI=Confidence interval.

**Fig. 3:**
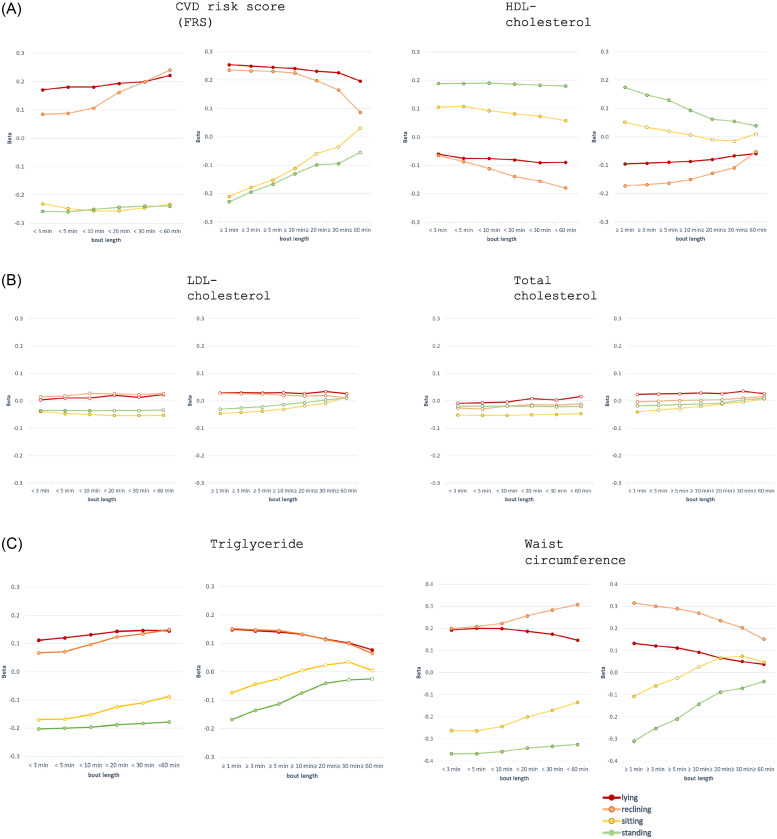
Associations between different bout lengths of stationary behavior and indicators of metabolic health (standardized beta coefficients). Colored circle indicates a statistically significant (*p* < 0.05) association, and open circle a non-significant association.

When the components of stationary behavior were analyzed in terms of <60 min bouts, more lying and reclining regardless of bout length were associated with higher CVD-score (FRS) (≤0.001), lower HDL-cholesterol (*p* < 0.001), higher triglyceride (*p* < 0.001), and larger waist circumference (*p* < 0.001) (Fig. 3, Supplementary Table 3). Greater sitting time accumulating from shorter than 30 min bouts was associated with lower CVD-score (FRS) (*p* < 0.001), higher HDL- (*p* < 0.001), lower LDL- (*p* = 0.004) and total cholesterol (*p* = 0.009), lower triglyceride (*p* < 0.001), and narrower waist circumference (*p* < 0.001). Greater sitting time accumulating from the bouts exceeding 20 min at a time was associated with a larger waist circumference (*p* < 0.001) indicating that health associations of sitting may depend on bout length. More standing regardless of bout length was associated with lower CVD-score (FRS) (≤0.001), higher HDL-cholesterol (*p* < 0.001), and smaller waist circumference (*p* < 0.001). The bout length exceeding 60 min gave more varying results (Fig. 3, Supplementary Table 3). The number of long bouts were scarce, especially regarding sitting and standing, which increases the confidence intervals.

## 4 Discussion

To our knowledge this is the first study to report on the relationship between different components of stationary behavior during waking hours and CVD risk score (FRS) as well as individual cardiometabolic risk factors in large population sample of working-aged adults. The analysis considered also MVPA levels. The novelty of the present study is that the components of stationary behavior were analyzed separately from each other, and the health associations of standing were assessed independently of those of lying, reclining and sitting.

The key results of the study showed that lying and reclining had positive associations with the CVD-score and several CVD risk factors while short sitting bouts and standing had negative associations, mainly regardless of MVPA level. These findings suggest that the associations between the different components of stationary behavior and several CVD risk factors (FRS, HDL-cholesterol, triglycerides, and waist circumference) are independent of the MVPA level. Further, lying and reclining, regardless of the bout length, were associated with poorer cardiometabolic health while especially short sitting bouts and standing were associated with indicators of better health. Thus, in studies evaluating the health associations, the components of stationary behavior (lying, reclining, sitting and standing) should be measured separately from each other, and especially sitting should be analyzed in different bout lengths.

Previously it has been stated that both the reduction of prolonged SB and its frequent interruption have resulted in benefits regarding several CVD risk factors including fasting glucose, insulin, HbA1c, HDL-concentrations, systolic blood pressure and vascular function [4]. Interrupting SB with short standing bouts has also been shown to be associated with better perceived health, vitality, and work performance [35]. However, total standing time seem not to be associated with a higher CVD risk, while there is a linear association for sitting time when the daily duration exceeds 10 h/day [18]. In many studies, standing has not been analyzed separately from SB or light PA, which may have blunted the health associations found.

Considering different bout lengths of SB, standing, and stepping Verswijveren et al. [20] studied the associations with cardiometabolic health markers using a thigh-worn device. SB (≥10, ≥30, and ≥60 min) and standing (≥10 and ≥30 min) bouts were detrimentally associated with BMI, percentage of body fat, fat mass, fat-free mass, lipid markers, and fasting glucose. The SB bouts exceeding 60 min and standing bouts exceeding 30 min had a larger influence than shorter bouts. In the present study, where standing, sitting, reclining and lying were analyzed separately, short <20 min sitting bouts were positively and longer bouts negatively associated with CVD risk factors, while standing was positively associated with cardiometabolic risk factors regardless of bout length. Thus, even shorter sitting bouts than the ones reported by Verswijveren et al. [20] may be unfavorable for health. Sitting posture seems to be a transitional zone regarding cardiometabolic health outcomes. Short sitting bouts may partly come from breaks during PA, which may partly explain positive health associations seen in the present results. However, the exact definition of a short or long bout remains vague and calls for further studies.

Regarding overweight as an individual risk factor Voigt et al. [24] reported that daily number of sedentary bouts lasting from 1 to 10 min were significantly associated with lower BMI but not with smaller waist circumference, and the number of bouts lasting >30 min was associated with larger waist circumference but not with BMI [24]. Thus, short and long SB bouts seem to have somewhat different associations with body composition although the definition of SB used by Voigt et al. [24] summarized all components with <100 counts per minute without considering body posture as an indicator of stationary behavior. However, the findings of Voigt et al. [24] are in accordance with the present study regarding the association between sitting and several health indicators.

In the present study, standing was assessed as an independent part of stationary behavior when a participant was in a standing position without movement [8]. This kind of activity is typical for performing many desk-based tasks. Sitting and standing seem to be more prevalent types of stationary behavior during typical office hours, while reclining and lying are more prevalent during evening hours, before time in bed [15]. Studying occupational sitting and standing Smith et al. [36] reported that occupations that involve predominantly standing were associated with a 2-fold risk of incident heart disease, compared with predominantly sitting occupations. In another study, there was evidence that standing for prolonged periods did not affect CVD mortality but at the same time increased the risk of orthostatic vascular disease, indicating an important deleterious consequence from longer standing periods [18].

An overview of systematic reviews including both self-reported and device-based studies reported that the context of SB may matter for health associations. TV viewing seems to be most consistently associated with unfavorable health outcomes, while computer and Internet use have even been suggested to be beneficial for cognitive function [37]. Further, a recent systematic review based on studies using Mendelian randomization showed that different types of SB have distinct causal effects on health outcomes, the leisure time TV watching increasing the risk for cardiovascular and metabolic conditions and the leisure time computer use decreasing the risk for cardiovascular diseases, musculoskeletal conditions, and Alzheimer’s disease [16]. However, this review provided no robust evidence on the association between the accelerometry-measured SB and health outcomes [16]. Differences in the associations may be due to different co-behaviors (e.g., snacking), muscle activity, and mental engagement between the types of SB [16]. This further emphasizes the present findings that the components of SB should not be combined as a single indicator of SB.

In the present study MVPA seemed not to have a role in the cardiometabolic health associations of lying reclining, sitting or standing, although PA can be associated with the reductions in LDL and total cholesterol [38]. Lack of effect in the present findings may be due that MVPA is a different behavior from stationary behavior. An individual can have large amounts of stationary behavior regardless of his/her MVPA level. Further, even in individuals who do not exercise or accumulate MVPA, daily activities detected by accelerometer have been associated with reductions in cardiovascular events including mortality [19].

The study includes a few limitations: First, we were unable to control for all potential confounders. Seasonal variation and exclusion of water-based activities were considered as potential sources of bias. Effect of seasonal variation was addressed by collecting data over several months during each study period. Potential bias due to exclusion of water-based activities was considered small since these activities are not among the most frequently performed activities among Finnish adults [39]. Further, the data sets used for the present analysis did not include dietary factors. The final analyses were adjusted only for age and sex. However, we conducted the analysis also by adjusting for marital status, education level, smoking status, self-reported diabetes status and self-reported hypertension, but these further adjustments did not affect the association between the components of stationary behavior and cardiometabolic risk factors. Second, the analysis is based on cross-sectional design which does not allow any causal interpretation of the findings. Third, the outcome variables were based on blood samples or self-reported health data, but no register-based data on CVD medications or events was available. Fourth, only less than half of the sample was reached, and just over half of the reached persons agreed to participate in the study, which indicates selective participation and consequent bias [6]. The study participants were slightly more educated than the general population in Finland [40], which may have affected the present findings. Fifth, the assessment of stationary behavior was based only on total times and different bout lengths of the activities, whereas no contextual data was available. Mentally active and passive forms of SB might have opposing relationships with health outcomes [37] which could not be considered in the present study. The present study used FRS as an indication of CVD risk. However, stationary behavior may form differential risk for CVD and for heart failure, and thus it would be important to add also a risk score for heart failure to future studies. Further, it would be important to assess potential longitudinal associations between stationary behavior and inflammatory markers, since there is evidence that SB and inflammatory markers are cross-sectionally associated with each other [41] and, inflammatory markers may mediate health deteriorating effects of SB [42,43].

The strengths of the present study are the comprehensive 24/7 measurement of physical behaviors (TIB, stationary behavior, and physical activity) and the large population-based samples of seven urban and suburban areas covering a wide age range of adults aged 20–69 years. The main strength of the study is that different components and several bout lengths of stationary behavior were analyzed with valid methods to assess body posture, and the components were used to analyze their associations with cardiometabolic health indicators. In the future, these components and bout lengths and their associations with health indicators should be analyzed in longitudinal designs.

## 5 Conclusions

Different components of stationary behavior had varying associations with CVD risk factors: Lying and reclining had negative associations with the CVD-score and HDL-cholesterol, triglyceride levels and wait circumference while short sitting bouts and standing had positive associations. MVPA level did not affect the associations between the components of stationary behavior and indicators of cardiometabolic health. The components of stationary behavior should be measured separately from each other, and the evaluation of different bout lengths should be considered, especially when assessing the health associations of sitting.

**Figure:**
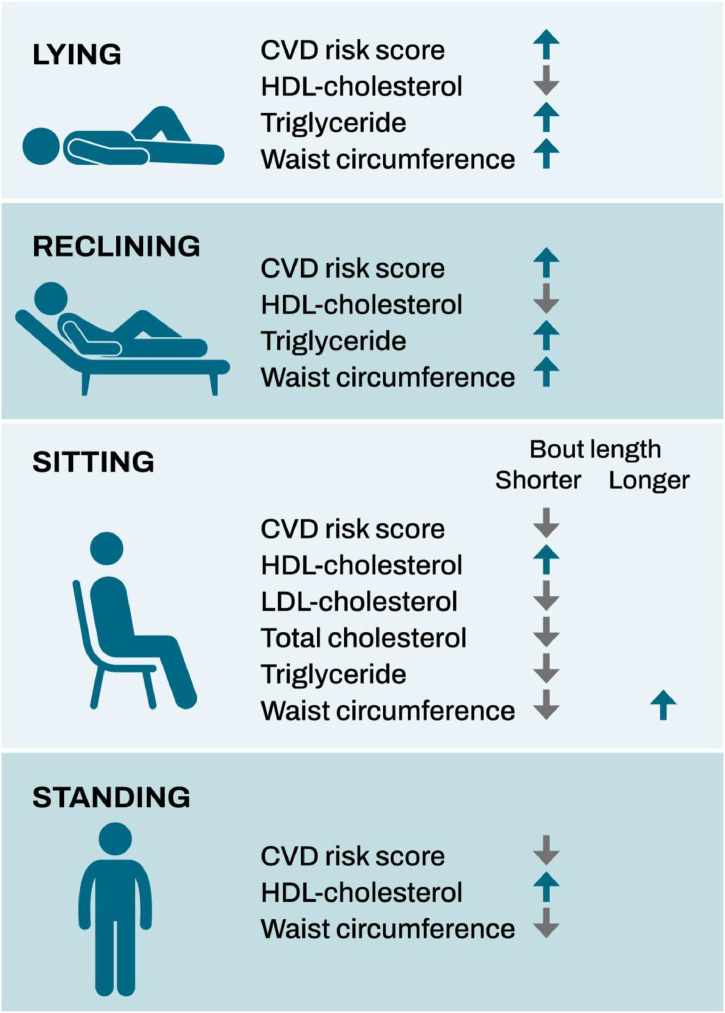
Central illustration. Summary of the results: Associations of different components of stationary behavior with cardiovascular disease risk score and several risk factors.

## Funding

This research was funded by the Finnish Ministry of Education and Culture.

## Ethics approval and consent to participate

This study was conducted in accordance with the Declaration of Helsinki and approved by the Regional Ethics Committee of the Expert Responsibility Area of Tampere University Hospital (R17030 and R2105). All study participants gave written informed consent before participating.

**Availability of data and materials**: The data are maintained at the UKK Institute. The datasets analyzed in the present study are not publicly available due to ethical restrictions (the Regional Ethics Committee of the Expert Responsibility Area of Tampere University Hospital), but more detailed information on the data is available from the corresponding author (TV) on reasonable request.

## CRediT authorship contribution statement

**Pauliina Husu:** Writing – original draft, Methodology, Investigation, Conceptualization. **Henri Vähä-Ypyä:** Writing – review & editing, Methodology, Data curation. **Kari Tokola:** Writing – review & editing, Formal analysis, Data curation. **Harri Sievänen:** Writing – review & editing, Supervision. **Onni Niemelä:** Writing – original draft, Methodology. **Tommi Vasankari:** Writing – review & editing, Supervision, Resources, Methodology, Funding acquisition, Conceptualization.

## Declaration of competing interest

The authors declare that they have no known competing financial interests or personal relationships that could have appeared to influence the work reported in this paper.

## Appendix Supplementary materials

Image, application 1
