# Supplementary material for: Greater amount of lying and reclining associate with cardiovascular disease risk score and several risk factors, while short sitting bouts and standing have opposite relation
Source: Am J Prev Cardiol. 2025 Oct 9;24:101327. doi: 10.1016/j.ajpc.2025.101327 (PMC12554051; doi:10.1016/j.ajpc.2025.101327)
Supplement: Supplementary file 1 [file mmc1.docx]

| Supplementary table 1A. CVD risk score (SCORE2) of the participants and p-value for sex difference. | | | | | |
| --- | --- | --- | --- | --- | --- |
|  |  |  |  |  |  |
|  |  | Men | Women | p-value* | Total |
| CVD risk score (SCORE2) | Mean (SD) | 6.6 (3.9) | 3.3 (2.6) | < 0.001 | 4.6 (3.6) |
| * 2-sided p-value from independent samples T-test | | |  |  |  |

| Supplementary table 1B. Associations between components of stationary behavior and CVD risk score (SCORE2) | | | | | | |
| --- | --- | --- | --- | --- | --- | --- |
| taking moderate-to-vigorous physical activity into account. | | | |  |  |  |
|  |  |  | Beta* | 95% CI | p-value |  |
| CVD risk score (SCORE2) | SB, lying | low MVPA | 0.19 | 0.06;0.33 | 0.005 |  |
|  |  | middle MVPA | 0.27 | 0.13;0.41 | <0.001 |  |
|  |  | high MVPA | 0.14 | 0.00;0,27 | 0.042 |  |
|  |  | total | 0.22 | 0.14;0.29 | <0.001 |  |
|  | SB, reclining | low MVPA | 0.18 | 0.05;0.31 | <0.001 |  |
|  |  | middle MVPA | 0.24 | 0.10;0.38 | 0.001 |  |
|  |  | high MVPA | 0.23 | 0.11;0,36 | <0.001 |  |
|  |  | total | 0.26 | 0.18;0.33 | <0.001 |  |
|  | SB, sitting | low MVPA | -0.23 | -0.37;-0.10 | 0.001 |  |
|  |  | middle MVPA | -0.28 | -0.42;-0.14 | <0.001 |  |
|  |  | high MVPA | -0.16 | -0.29;-0.02 | 0.021 |  |
|  |  | total | -0.19 | -0.27;-0.11 | <0.001 |  |
|  | Standing | low MVPA | -0.17 | -0.30;-0.04 | 0.012 |  |
|  |  | middle MVPA | -0.29 | -0.43;-0.15 | <0.001 |  |
|  |  | high MVPA | -0.20 | -0.33;-0.07 | 0.002 |  |
|  |  | total | -0.21 | -0.28;-0,13 | <0.001 |  |
| * standardized beta coefficients | |  |  |  |  |  |
| CVD=cardiovascular disease, SB=sedentary behavior, MVPA=moderate-to-vigorous physical activity, CI=confidence interval | | | | | | |

| Supplementary table 1C. Associations between different bout lengths of stationary behavior and | | | | | |
| --- | --- | --- | --- | --- | --- |
| CVD risk score (SCORE2). |  |  |  |  |  |
|  |  | CVD risk score (SCORE2) | | |  |
|  | bout length | B* | 95% CI | p-value |  |
| Lying | <3 min | 0.20 | 0.10;0.29 | **<0.001** |  |
|  | <5 min | 0.35 | 0.19;0.50 | **<0.001** |  |
|  | <10 min | 0.59 | 0.30;0.87 | **<0.001** |  |
|  | <20 min | 0.96 | 0.48;1.43 | **<0.001** |  |
|  | <30 min | 1.33 | 0.71;1.94 | **<0.001** |  |
|  | <60 min | 2.21 | 1.36;3.05 | **<0.001** |  |
|  |  |  |  |  |  |
|  | ≥60 min | 0.70 | 0.31;1.09 | **<0.001** |  |
|  | ≥30 min | 1.59 | 0.95;2.21 | **<0.001** |  |
|  | ≥20 min | 1.95 | 1.19;2.71 | **<0.001** |  |
|  | ≥10 min | 2.32 | 1.43;3.21 | **<0.001** |  |
|  | ≥5 min | 2.56 | 1.59;3.53 | **<0.001** |  |
|  | ≥3 min | 2.71 | 1.71;3.71 | **<0.001** |  |
|  | ≥1 min | 2.87 | 1.83;3.90 | **<0.001** |  |
| Reclining | <3 min | 0.22 | -0.03;0.48 | 0.089 |  |
|  | <5 min | 0.41 | -0.01;0.83 | 0.054 |  |
|  | <10 min | 0.84 | 0.13;1.56 | **0.021** |  |
|  | <20 min | 2.26 | 1.18;3.35 | **<0.001** |  |
|  | <30 min | 3.38 | 2.06;4.71 | **<0.001** |  |
|  | <60 min | 5.80 | 4.14;7.47 | **<0.001** |  |
|  |  |  |  |  |  |
|  | ≥60 min | 0.92 | 0.19;1.66 | **0.014** |  |
|  | ≥30 min | 3.34 | 2.13;4.56 | **<0.001** |  |
|  | ≥20 min | 4.46 | 3.03;5.90 | **<0.001** |  |
|  | ≥10 min | 5.88 | 4.17;7.59 | **<0.001** |  |
|  | ≥5 min | 6.32 | 4.48;8.16 | **<0.001** |  |
|  | ≥3 min | 6.50 | 4.60;8.40 | **<0.001** |  |
|  | ≥1 min | 6.50 | 4.71;8.59 | **<0.001** |  |
| Sitting | <3 min | -0.83 | -1.18;-0.48 | **<0.001** |  |
|  | <5 min | -1.37 | -1.89;-0.85 | **<0.001** |  |
|  | <10 min | -2.23 | -3.01;-1.45 | **<0.001** |  |
|  | <20 min | -2.94 | -3.99;-1.89 | **<0.001** |  |
|  | <30 min | -3.14 | -4.32;-1.96 | **<0.001** |  |
|  | <60 min | -3.40 | -4.73;-2.07 | **<0.001** |  |
|  |  |  |  |  |  |
|  | ≥60 min | 0.05 | -0.15;0.25 | 0.651 |  |
|  | ≥30 min | -0.21 | -0.66;0.23 | 0.341 |  |
|  | ≥20 min | -0.41 | -1.02;0.20 | 0.189 |  |
|  | ≥10 min | -1.12 | -2.04;-0.21 | **0.016** |  |
|  | ≥5 min | -1.98 | -3.12;-0.85 | **0.001** |  |
|  | ≥3 min | -2.52 | -3.76;-1.27 | **<0.001** |  |
|  | ≥1 min | -3.17 | -4.53;-1.80 | **<0.001** |  |
| Standing | <3 min | -1.26 | -1.70;-0.82 | **<0.001** |  |
|  | <5 min | -1.85 | -2.48;-1.22 | **<0.001** |  |
|  | <10 min | -2.54 | -3.42;-1.67 | **<0.001** |  |
|  | <20 min | -2.99 | -4.05;-1.93 | **<0.001** |  |
|  | <30 min | -3.13 | -4.25;-2.00 | **<0.001** |  |
|  | <60 min | -3.29 | -4.48;-2.10 | **<0.001** |  |
|  |  |  |  |  |  |
|  | ≥60 min | -0.01 | -0.10;0.08 | 0.832 |  |
|  | ≥30 min | -0.17 | -0.40;0.05 | 0.135 |  |
|  | ≥20 min | -0.31 | -0.64;0.02 | 0.063 |  |
|  | ≥10 min | -0.76 | -1.30;-0.21 | **0.007** |  |
|  | ≥5 min | -1.46 | -2.23;-0.69 | **<0.001** |  |
|  | ≥3 min | -2.04 | -2.96;-1.12 | **<0.001** |  |
|  | ≥1 min | -2.99 | -4.14;-1.85 | **<0.001** |  |
| *non-standardized regression coefficient | |  |  |  |  |
| CVD=cardiovascular disease, CI=confidence interval | | |  |  |  |

| Supplementary table 2. Mean differences of the stationary bouts between the age groups | | | | | | | | |  |  |
| --- | --- | --- | --- | --- | --- | --- | --- | --- | --- | --- |
| with statistical significances. | | |  |  |  |  |  |  |  |  |
|  |  |  |  |  | Men |  |  |  | Women |  |
| Stationary behavior | Bout length | Age group |  | Mean difference | 95% CI | p-value |  | Mean difference | 95% CI | p-value |
| Lying | <10 min | 20-29 | 30-39 | 3.06 | -0.51;6.63 | 0.093 |  | **4.32** | **2.17;6.47** | **<0.001** |
|  |  |  | 40-49 | **6.68** | **3.23;10.13** | **<0.001** |  | **6.46** | **4.4;8.52** | **<0.001** |
|  |  |  | 50-59 | **5.42** | **2.06;8.77** | **0.002** |  | **6.83** | **4.81;8.85** | **<0.001** |
|  |  |  | 60-69 | **4.97** | **1.71;8.22** | **0.003** |  | **4.79** | **2.81;6.77** | **<0.001** |
|  |  | 30-39 | 40-49 | **3.62** | **1.08;6.15** | **0.005** |  | **2.14** | **0.44;3.83** | **0.013** |
|  |  |  | 50-59 | 2.35 | -0.07;4.77 | 0.056 |  | **2.51** | **0.85;4.16** | **0.003** |
|  |  |  | 60-69 | 1.90 | -0.37;4.17 | 0.100 |  | 0.47 | -1.13;2.07 | 0.564 |
|  |  | 40-49 | 50-59 | -1.36 | -3.49;0.96 | 0.266 |  | 0.37 | -1.16;1.9 | 0.637 |
|  |  |  | 60-69 | -1.72 | -3.78;0.35 | 0.103 |  | **-1.67** | **-3.14;-0.2** | **0.026** |
|  |  | 50-59 | 60-69 | -0.45 | -2.36;1.46 | 0.643 |  | **-2.04** | **-3.46;-0.61** | **0.005** |
|  | 10-19.9 min | 20-29 | 30-39 | 2.09 | -0.77;4.95 | 0.151 |  | **2.62** | **0.84;4.4** | **0.004** |
|  |  |  | 40-49 | 3.31 | 0.55;6.07 | 0.019 |  | **4.07** | **2.37;5.77** | **<0.001** |
|  |  |  | 50-59 | **4.18** | **1.49;6.86** | **0.002** |  | **5.05** | **3.38;6.72** | **<0.001** |
|  |  |  | 60-69 | **3.41** | **0.81;6.01** | **0.010** |  | **4.67** | **3.04;6.31** | **<0.001** |
|  |  | 30-39 | 40-49 | 1.22 | -0.81;3.25 | 0.240 |  | **1.45** | **0.05;2.85** | **0.043** |
|  |  |  | 50-59 | 2.08 | 0.15;4.02 | 0.035 |  | **2.43** | **1.06;3.8** | **0.001** |
|  |  |  | 60-69 | 1.32 | -0.49;3.13 | 0.154 |  | **2.06** | **0.73;3.38** | **0.002** |
|  |  | 40-49 | 50-59 | 0.87 | -0.92;2.65 | 0.341 |  | 0.98 | -0.28;2.25 | 0.129 |
|  |  |  | 60-69 | 0.10 | -1.55;1.75 | 0.904 |  | 0.61 | -0.61;1.82 | 0.329 |
|  |  | 50-59 | 60-69 | -0.76 | -2.30;0.77 | 0.327 |  | -0.38 | -1.55;0.8 | 0.532 |
|  | 20-29.9 min | 20-29 | 30-39 | 1.85 | -0.63;4.34 | 0.143 |  | **2.09** | **0.58;3.59** | **0.007** |
|  |  |  | 40-49 | 1.69 | -0.71;4.09 | 0.167 |  | **2.44** | **1;3.88** | **0.001** |
|  |  |  | 50-59 | 1.23 | -1.10;3.57 | 0.301 |  | **2.91** | **1.5;4.33** | **<0.001** |
|  |  |  | 60-69 | 0.47 | -1.79;2.73 | 0.682 |  | **1.61** | **0.22;2.99** | **0.023** |
|  |  | 30-39 | 40-49 | -0.16 | -1.93;1.60 | 0.857 |  | 0.35 | -0.84;1.54 | 0.564 |
|  |  |  | 50-59 | -0.62 | -2.30;1.06 | 0.469 |  | 0.83 | -0.33;1.98 | 0.163 |
|  |  |  | 60-69 | -1.38 | -2.96;0.19 | 0.086 |  | -0.48 | -1.6;0.64 | 0.400 |
|  |  | 40-49 | 50-59 | -0.46 | -2.01;1.09 | 0.561 |  | 0.48 | -0.6;1.55 | 0.384 |
|  |  |  | 60-69 | -1.22 | -2.65;0.22 | 0.096 |  | -0.83 | -1.86;0.2 | 0.114 |
|  |  | 50-59 | 60-69 | -0.76 | -2.09;0.57 | 0.262 |  | **-1.31** | **-2.3;-0.31** | **0.010** |
|  | 30-59.9 min | 20-29 | 30-39 | 0.46 | -4.15;5.06 | 0.845 |  | 1.59 | -0.92;4.11 | 0.214 |
|  |  |  | 40-49 | -1.61 | -6.06;2.83 | 0.477 |  | 2.98 | 0.58;5.39 | 0.015 |
|  |  |  | 50-59 | -3.76 | -8.09;0.57 | 0.089 |  | 1.74 | -0.63;4.11 | 0.149 |
|  |  |  | 60-69 | **-5.29** | **-9.49;-1.10** | **0.013** |  | -0.25 | -2.56;2.07 | 0.835 |
|  |  | 30-39 | 40-49 | -2.07 | -5.35;1.20 | 0.215 |  | 1.39 | -0.59;3.37 | 0.169 |
|  |  |  | 50-59 | **-4.22** | **-7.33;-1.10** | **0.008** |  | 0.15 | -1.79;2.08 | 0.882 |
|  |  |  | 60-69 | **-5.75** | **-8.68;-2.83** | **<0.001** |  | -1.84 | -3.71;0.03 | 0.054 |
|  |  | 40-49 | 50-59 | -2.15 | -5.02;0.73 | 0.143 |  | -1.24 | -3.03;0.54 | 0.173 |
|  |  |  | 60-69 | **-3.68** | **-6.34;-1.02** | **0.007** |  | **-3.23** | **-4.95;-1.51** | **<0.001** |
|  |  | 50-59 | 60-69 | -1.54 | -4.00;0.93 | 0.222 |  | -1.99 | -2.07;2.56 | 0.835 |
|  | >60 min | 20-29 | 30-39 | 0.23 | -4.67;5.14 | 0.925 |  | 1.42 | -1.43;4.26 | 0.329 |
|  |  |  | 40-49 | -0.64 | -5.37;4.10 | 0.791 |  | 0.88 | -1.84;3.6 | 0.525 |
|  |  |  | 50-59 | -3.61 | -8.23;1.00 | 0.125 |  | 0.70 | -1.98;3.37 | 0.609 |
|  |  |  | 60-69 | -3.89 | -8.36;0.58 | 0.088 |  | -1.21 | -3.82;1.41 | 0.366 |
|  |  | 30-39 | 40-49 | -0.87 | -4.36;2.61 | 0.623 |  | -0.53 | -2.78;1.71 | 0.641 |
|  |  |  | 50-59 | **-3.85** | **-7.17;-0.53** | **0.023** |  | -0.72 | -2.91;1.47 | 0.520 |
|  |  |  | 60-69 | **-4.12** | **-7.24;-1.01** | **0.009** |  | **-2.62** | **-4.74;-0.51** | **0.015** |
|  |  | 40-49 | 50-59 | -2.97 | -6.04;0.09 | 0.057 |  | -0.18 | -2.21;1.84 | 0.858 |
|  |  |  | 60-69 | **-3.25** | **-6.08;-0.41** | **0.025** |  | **-2.09** | **-4.03;-0.15** | **0.035** |
|  |  | 50-59 | 60-69 | -0.27 | -2.90;2.35 | 0.838 |  | **-1.90** | **-3.79;-0.02** | **0.047** |
| Reclining | <10 min | 20-29 | 30-39 | **8.53** | **0.48;16.58** | **0.038** |  | 4.60 | -0.52;9.72 | 0.078 |
|  |  |  | 40-49 | **14.05** | **6.28;21.81** | **<0.001** |  | **8.90** | **4.01;13.79** | **<0.001** |
|  |  |  | 50-59 | **15.48** | **7.91;23.05** | **<0.001** |  | **10.18** | **5.37;15** | **<0.001** |
|  |  |  | 60-69 | **12.57** | **5.24;19.89** | **0.001** |  | **5.43** | **0.72;10.13** | **0.024** |
|  |  | 30-39 | 40-49 | 5.52 | -0.20;11.24 | 0.059 |  | **4.30** | **0.27;8.34** | **0.037** |
|  |  |  | 50-59 | **6.96** | **1.51;12.41** | **0.012** |  | **5.59** | **1.65;9.52** | **0.005** |
|  |  |  | 60-69 | 4.04 | 1.07;9.15 | 0.121 |  | 0.83 | -2.98;4.63 | 0.670 |
|  |  | 40-49 | 50-59 | 1.44 | -3.59;6.46 | 0.575 |  | 1.28 | -2.36;4.92 | 0.490 |
|  |  |  | 60-69 | -1.48 | -6.13;3.17 | 0.532 |  | -3.48 | -6.97;0.02 | 0.051 |
|  |  | 50-59 | 60-69 | -2.92 | -7.23;1,39 | 0.185 |  | **-4.76** | **-8.15;-1.37** | **0.006** |
|  | 10-19.9 min | 20-29 | 30-39 | 4.84 | -1.29;10.98 | 0.122 |  | 2.18 | -1.59;5.95 | 0.257 |
|  |  |  | 40-49 | 5.27 | -0.65;11.19 | 0.081 |  | 1.45 | -2.15;5.05 | 0.430 |
|  |  |  | 50-59 | **6.03** | **0.26;11,80** | **0.041** |  | 1.45 | -2.1;4.99 | 0.424 |
|  |  |  | 60-69 | 1.25 | -4.33;6.84 | 0.660 |  | -0.19 | -3.66;3.28 | 0.915 |
|  |  | 30-39 | 40-49 | 0.43 | -3.93;4.79 | 0.848 |  | -0.73 | -3.7;2.24 | 0.631 |
|  |  |  | 50-59 | 1.18 | -2.97;5.33 | 0.577 |  | -0.73 | -3.63;2.17 | 0.620 |
|  |  |  | 60-69 | -3.59 | -7.48;0.30 | 0.071 |  | -2.37 | -5.17;0.44 | 0.098 |
|  |  | 40-49 | 50-59 | 0.75 | -3.07;4.58 | 0.699 |  | -0.01 | -2.69;2.67 | 0.997 |
|  |  |  | 60-69 | **-4.02** | **-7.56;-0.47** | **0.026** |  | -1.64 | -4.21;0.94 | 0.212 |
|  |  | 50-59 | 60-69 | **-4.77** | **-8.06;-1.49** | **0.004** |  | -1.63 | -4.13;0.86 | 0.199 |
|  | 20-29.9 min | 20-29 | 30-39 | 4.23 | -0.58;9.03 | 0.085 |  | 0.44 | -2.6;3.48 | 0.776 |
|  |  |  | 40-49 | 0.31 | -4.32,4.95 | 0.894 |  | -2.46 | -5.36;0.45 | 0.098 |
|  |  |  | 50-59 | 0.54 | -3.98,5.05 | 0.816 |  | **-3.85** | **-6.71;-0.99** | **0.008** |
|  |  |  | 60-69 | **-8.04** | **-12.41;-3.66** | **<0.001** |  | **-8.13** | **-10.93;-5.33** | **<0.001** |
|  |  | 30-39 | 40-49 | **-3.91** | **-7.33;-0.50** | **0.025** |  | **-2.90** | **-5.29;-0.5** | **0.018** |
|  |  |  | 50-59 | **-3.69** | **-6.94;-0.44** | **0.026** |  | **-4.29** | **-6.63;-1.95** | **<0.001** |
|  |  |  | 60-69 | **-12.27** | **-15.31;-9.22** | **<0.001** |  | **-8.57** | **-10.83;-6.31** | **<0.001** |
|  |  | 40-49 | 50-59 | 0.22 | -2.78;3.22 | 0.885 |  | -1.40 | -3.56;0.77 | 0.205 |
|  |  |  | 60-69 | **-8.35** | **-11.13,-5.58** | **<0.001** |  | **-5.67** | **-7.75;-3.59** | **<0.001** |
|  |  | 50-59 | 60-69 | **-8.57** | **-11.15;-6.00** | **<0.001** |  | **-4.28** | **-6.29;-2.26** | **<0.001** |
|  | 30-59.9 min | 20-29 | 30-39 | 4.04 | -4.29;12.38 | 0.342 |  | 1.07 | -4.02;6.15 | 0.681 |
|  |  |  | 40-49 | 0.54 | 7.50;8.59 | 0.894 |  | **-7.20** | **-12.06;-2.34** | **0.004** |
|  |  |  | 50-59 | -6.63 | 14.47,1.20 | 0.097 |  | **-9.98** | **-14.76;-5.19** | **<0.001** |
|  |  |  | 60-69 | **-22.09** | **29.68;-14.50** | **<0.001** |  | **-22.63** | **-27.3;-17.95** | **<0.001** |
|  |  | 30-39 | 40-49 | -3.50 | -9.42;2.43 | 0.247 |  | **-8.27** | **-12.27;-4.26** | **<0.001** |
|  |  |  | 50-59 | **-10.68** | **-16.32;-5.03** | **<0.001** |  | **-11.04** | **-14.95;-7.13** | **<0.001** |
|  |  |  | 60-69 | **-26.13** | **-31.42;-20.84** | **<0.001** |  | **-23.69** | **-27.48;-19.91** | **<0.001** |
|  |  | 40-49 | 50-59 | **-7.18** | **-12.38,-1.98** | **0.007** |  | -2.77 | -6.39;0.84 | 0.132 |
|  |  |  | 60-69 | **-22.64** | **27.45;-17.82** | **<0.001** |  | **-15.43** | **-18.9;-11.95** | **<0.001** |
|  |  | 50-59 | 60-69 | **-15.46** | **-19.92;-10.99** | **<0.001** |  | **-12.65** | **-16.02;-9.29** | **<0.001** |
|  | >60 min | 20-29 | 30-39 | 7.37 | -1.06;15.80 | 0.087 |  | 0.39 | -4.69;5.46 | 0.880 |
|  |  |  | 40-49 | **10.25** | **2.11;18.39** | **0.014** |  | **-5.73** | **-10.58;-0.88** | **0.021** |
|  |  |  | 50-59 | 4.02 | -3.91;11.95 | 0.320 |  | **-10.06** | **-14.83;-5.29** | **<0.001** |
|  |  |  | 60-69 | -4.32 | -12.00;3.35 | 0.269 |  | **-14.43** | **-19.09;-9.76** | **<0.001** |
|  |  | 30-39 | 40-49 | 2.88 | -3.11;8.88 | 0.345 |  | **-6.12** | **-10.12;-2.12** | **0.003** |
|  |  |  | 50-59 | -3.34 | -9.05,2.36 | 0.251 |  | **-10.45** | **-14.36;-6.55** | **<0.001** |
|  |  |  | 60-69 | **-11.69** | **-17.04;-6.34** | **<0.001** |  | **-14.82** | **-18.59;-11.04** | **<0.001** |
|  |  | 40-49 | 50-59 | **-6.23** | **-11.49;-0.97** | **0.020** |  | **-4.33** | **-7.94;-0.72** | **0.019** |
|  |  |  | 60-69 | **-14.58** | **-19.45;-9.70** | **<0.001** |  | **-8.70** | **-12.16;-5.23** | **<0.001** |
|  |  | 50-59 | 60-69 | **-8.35** | **-12.86;-3.83** | **<0.001** |  | **-4.37** | **-7.72;-1.01** | **0.011** |
| Sitting | <10 min | 20-29 | 30-39 | -0.06 | -8.33;8.21 | 0.989 |  | -2.63 | -8.24;2.99 | 0.359 |
|  |  |  | 40-49 | 0.44 | -7.54;8.43 | 0.913 |  | 0.93 | -4.43;6.3 | 0.733 |
|  |  |  | 50-59 | 3.30 | -4.48;11.07 | 0.406 |  | 3.57 | -1.71;8.85 | 0.185 |
|  |  |  | 60-69 | **10.44** | **2.91;17.97** | **0.007** |  | **6.23** | **1.07;11.39** | **0.018** |
|  |  | 30-39 | 40-49 | 0.50 | -5.38;6.38 | 0.867 |  | 3.56 | -0.86;7.98 | 0.115 |
|  |  |  | 50-59 | 3.36 | -2-24;8.96 | 0.240 |  | 6.19 | 1.87;10.51 | 0.005 |
|  |  |  | 60-69 | **10.50** | **5.25;15.75** | **<0.001** |  | **8.86** | **4.68;13.03** | **<0.001** |
|  |  | 40-49 | 50-59 | 2.85 | -2.31;8.02 | 0.278 |  | 2.63 | -1.36;6.62 | 0.196 |
|  |  |  | 60-69 | **10.00** | **5.22;14.78** | **<0.001** |  | **5.30** | **1.46;9.13** | **0.007** |
|  |  | 50-59 | 60-69 | **7.15** | **2.72:11.58** | **0.002** |  | 2.66 | -1.05;6.38 | 0.160 |
|  | 10-19.9 min | 20-29 | 30-39 | 0.04 | -4.53;4.61 | 0.987 |  | 2.54 | -0.39;5.47 | 0.089 |
|  |  |  | 40-49 | -2.42 | -6.83;1.99 | 0.282 |  | 0.99 | -1.81;3.79 | 0.489 |
|  |  |  | 50-59 | 0.95 | -3.34;5.25 | 0.664 |  | -0.07 | -2.82;2.68 | 0.961 |
|  |  |  | 60-69 | 3.57 | -0.59,7,73 | 0.092 |  | **3.79** | **1.09;6.48** | **0.006** |
|  |  | 30-39 | 40-49 | -2.46 | -5.70;0.79 | 0.138 |  | -1.55 | -3.86;0.75 | 0.187 |
|  |  |  | 50-59 | 0.91 | -2.18;4.01 | 0.562 |  | **-2.61** | **-4.86;-0.36** | **0.023** |
|  |  |  | 60-69 | **3.54** | **0.64,6.43** | **0.017** |  | 1.25 | -0.93;3.42 | 0.263 |
|  |  | 40-49 | 50-59 | **3.37** | **0.52;6.22** | **0.021** |  | -1.06 | -3.14;1.03 | 0.320 |
|  |  |  | 60-69 | **5.99** | **3.35,8.63** | **<0.001** |  | **2.80** | **0.8;4.8** | **0.006** |
|  |  | 50-59 | 60-69 | **2.62** | **0.17;5.07** | **0.036** |  | **3.85** | **1.92;5.79** | **<0.001** |
|  | 20-29.9 min | 20-29 | 30-39 | 0.63 | -2.19;3.45 | 0.661 |  | 0.76 | -1.08;2.6 | 0.421 |
|  |  |  | 40-49 | 0.21 | -2.51;2.93 | 0.879 |  | 0.03 | -1.73;1.79 | 0.972 |
|  |  |  | 50-59 | -0.03 | -2.68;2.63 | 0.985 |  | -1.39 | -3.12;0.34 | 0.115 |
|  |  |  | 60-69 | 1.43 | -1.13;3.99 | 0.275 |  | 0.42 | -1.27;2.11 | 0.628 |
|  |  | 30-39 | 40-49 | -0.42 | -2.42;1.59 | 0.682 |  | -0.72 | -2.17;0.72 | 0.327 |
|  |  |  | 50-59 | -0.66 | -2.56;1.25 | 0.500 |  | **-2.15** | **-3.56;-0.73** | **0.003** |
|  |  |  | 60-69 | 0.80 | -0.99;2.59 | 0.382 |  | -0.34 | -1.71;1.03 | 0.628 |
|  |  | 40-49 | 50-59 | -0.24 | -2.00;1.52 | 0.791 |  | -1.42 | -2.73;-0.11 | 0.033 |
|  |  |  | 60-69 | 1.22 | -0.41;2.85 | 0.143 |  | 0.39 | -0.87;1.64 | 0.546 |
|  |  | 50-59 | 60-69 | 1.45 | -0.06;2.96 | 0.059 |  | **1.81** | **0.59;3.03** | **0.004** |
|  | 30-59.9 min | 20-29 | 30-39 | -0.62 | -3.98;2.74 | 0.716 |  | 0.60 | -1.75;2.95 | 0.618 |
|  |  |  | 40-49 | -1.27 | -4.51;1.97 | 0.442 |  | -0.50 | -2.75;1.74 | 0.660 |
|  |  |  | 50-59 | -0.23 | 3.39;2.93 | 0.886 |  | -1.10 | -3.31;1.12 | 0.331 |
|  |  |  | 60-69 | -0.83 | -.3.88,2.23 | 0.597 |  | -0.14 | -2.3;2.03 | 0.901 |
|  |  | 30-39 | 40-49 | -0.65 | -3.03;1.74 | 0.596 |  | -1.10 | -2.96;0.75 | 0.243 |
|  |  |  | 50-59 | 0.39 | -1.88;2.67 | 0.735 |  | -1.70 | -3.51;0.11 | 0.066 |
|  |  |  | 60-69 | -0.20 | -2.33;1.93 | 0.853 |  | -0.74 | -2.49;1.01 | 0.410 |
|  |  | 40-49 | 50-59 | 1.04 | -1.06;3.14 | 0.331 |  | -0.59 | -2.27;1.08 | 0.486 |
|  |  |  | 60-69 | 0.44 | -1.5;2.39 | 0.653 |  | 0.37 | -1.24;1.98 | 0.654 |
|  |  | 50-59 | 60-69 | -0.59 | -2.39;1.21 | 0.517 |  | 0.96 | -0.6;2.52 | 0.226 |
|  | >60 min | 20-29 | 30-39 | -1.03 | -3.35;1.29 | 0.385 |  | 0.55 | -1.15;2.24 | 0.527 |
|  |  |  | 40-49 | -0.24 | -2.48;1.99 | 0.831 |  | -0.39 | -2.01;1.22 | 0.633 |
|  |  |  | 50-59 | -0.43 | -2.61;1.75 | 0.700 |  | -1.11 | -2.71;0.48 | 0.170 |
|  |  |  | 60-69 | -0.15 | -2.27;1.96 | 0.887 |  | 0.33 | -1.22;1.89 | 0.673 |
|  |  | 30-39 | 40-49 | 0.78 | -0.87;2.43 | 0.352 |  | -0.94 | -2.27;0.39 | 0.167 |
|  |  |  | 50-59 | 0.60 | -0.97;2.17 | 0.455 |  | **-1.66** | **-2.96;-0.36** | **0.013** |
|  |  |  | 60-69 | 0.87 | -0.6;2.35 | 0.245 |  | -0.21 | -1.47;1.05 | 0.742 |
|  |  | 40-49 | 50-59 | -0.19 | -1.63;1.26 | 0.801 |  | -0.72 | -1.92;0.48 | 0.241 |
|  |  |  | 60-69 | 0.09 | -1.25;1.43 | 0.895 |  | 0.73 | -0.43;1.89 | 0.217 |
|  |  | 50-59 | 60-69 | 0.28 | -0.97;1.52 | 0.663 |  | **1.45** | **0.33;2.57** | **0.011** |
| Standing | <10 min | 20-29 | 30-39 | **-12.45** | **-20.57;-4.33** | **0.003** |  | **-11.01** | **-17.38;-4.64** | **0.001** |
|  |  |  | 40-49 | **-15.99** | **-23.83;-8.16** | **<0.001** |  | **-14.90** | **-20.99;-8.81** | **<0.001** |
|  |  |  | 50-59 | **-10.90** | **-18.54;-3.27** | **0.005** |  | **-9.37** | **-15.36;-3.38** | **0.002** |
|  |  |  | 60-69 | -2.18 | -9.57;5.21 | 0.563 |  | -2.18 | -8.03;3.68 | 0.466 |
|  |  | 30-39 | 40-49 | -3.54 | -9.31;2.23 | 0.230 |  | -3.89 | -8.9;1.13 | 0.129 |
|  |  |  | 50-59 | 1.55 | -3.95;7.05 | 0.580 |  | 1.64 | -3.26;6.54 | 0.512 |
|  |  |  | 60-69 | **10.28** | **5.12;15.43** | **<0.001** |  | **8.84** | **4.1;13.58** | **<0.001** |
|  |  | 40-49 | 50-59 | **5.09** | **0.02;10.15** | **0.049** |  | **5.53** | **1;10.05** | **0.017** |
|  |  |  | 60-69 | **13.81** | **9.12;18.5** | **<0.001** |  | **12.72** | **8.37;17.07** | **<0.001** |
|  |  | 50-59 | 60-69 | **8.73** | **4.38;13.07** | **<0.001** |  | **7.20** | **2.98;11.41** | **0.001** |
|  | 10-19.9 min | 20-29 | 30-39 | -2.51 | -5.02;0.01 | 0.051 |  | 0.10 | -1.83;2.04 | 0.918 |
|  |  |  | 40-49 | **-3.86** | **-6.28;-1.43** | **0.002** |  | -1.82 | -3.67;0.03 | 0.053 |
|  |  |  | 50-59 | **-3.60** | **-5.96;-1.23** | **0.003** |  | -0.87 | -2.69;0.95 | 0.351 |
|  |  |  | 60-69 | **-2.35** | **-4.64;-0.06** | **0.044** |  | 1.11 | -0.67;2.89 | 0.223 |
|  |  | 30-39 | 40-49 | -1.35 | -3.14;0.44 | 0.139 |  | **-1.93** | **-3.45;-0.4** | **0.013** |
|  |  |  | 50-59 | -1.09 | -2.79;0.61 | 0.210 |  | -0.97 | -2.46;0.52 | 0.203 |
|  |  |  | 60-69 | 0.15 | -1.44;1.75 | 0.849 |  | 1.00 | -0.44;2.44 | 0.172 |
|  |  | 40-49 | 50-59 | 0.26 | -1.31;1.83 | 0.744 |  | 0.96 | -0.42;2.33 | 0.173 |
|  |  |  | 60-69 | **1.50** | **0.05;2.96** | **0.043** |  | **2.93** | **1.61;4.25** | **<0.001** |
|  |  | 50-59 | 60-69 | 1.24 | -0.11;2.59 | 0.071 |  | **1.97** | **0.69;3.25** | **0.003** |
|  | 20-29.9 min | 20-29 | 30-39 | **-1.59** | **-2.88;-0.29** | **0.017** |  | -0.55 | -1.51;0.42 | 0.266 |
|  |  |  | 40-49 | **-1.75** | **-3;-0.5** | **0.006** |  | **-1.57** | **-2.49;-0.65** | **0.001** |
|  |  |  | 50-59 | -1.01 | -2.23;0.21 | 0.105 |  | **-1.10** | **-2.01;-0.19** | **0.018** |
|  |  |  | 60-69 | -0.99 | -2.17;0.19 | 0.101 |  | 0.12 | -0.77;1.01 | 0.792 |
|  |  | 30-39 | 40-49 | -0.16 | -1.09;0.76 | 0.729 |  | **-1.02** | **-1.78;-0.26** | **0.008** |
|  |  |  | 50-59 | 0.58 | -0.3;1.46 | 0.197 |  | -0.55 | -1.29;0.19 | 0.146 |
|  |  |  | 60-69 | 0.60 | -0.23;1.42 | 0.155 |  | 0.67 | -0.05;1.39 | 0.068 |
|  |  | 40-49 | 50-59 | 0.74 | -0.07;1.55 | 0.073 |  | 0.47 | -0.22;1.16 | 0.179 |
|  |  |  | 60-69 | **0.76** | **0.01;1.51** | **0.047** |  | **1.69** | **1.03;2.35** | **<0.001** |
|  |  | 50-59 | 60-69 | 0.02 | -0.67;0.71 | 0.955 |  | **1.22** | **0.58;1.86** | **<0.001** |
|  | 30-59.9 min | 20-29 | 30-39 | -0.82 | -2.41;0.77 | 0.312 |  | -0.49 | -1.62;0.63 | 0.389 |
|  |  |  | 40-49 | -1.24 | -2.78;0.29 | 0.112 |  | **-1.24** | **-2.31;-0.17** | **0.023** |
|  |  |  | 50-59 | -0.87 | -2.37;0.62 | 0.253 |  | **-1.28** | **-2.33;-0.22** | **0.018** |
|  |  |  | 60-69 | -0.34 | -1.79;1.11 | 0.644 |  | -0.08 | -1.11;0.96 | 0.886 |
|  |  | 30-39 | 40-49 | -0.42 | -1.55;0.71 | 0.462 |  | -0.75 | -1.63;0.14 | 0.098 |
|  |  |  | 50-59 | -0.05 | -1.13;1.03 | 0.925 |  | -0.78 | -1.65;0.08 | 0.075 |
|  |  |  | 60-69 | 0.48 | -0.53;1.49 | 0.352 |  | 0.42 | -0.42;1.25 | 0.327 |
|  |  | 40-49 | 50-59 | 0.37 | -0.62;1.36 | 0.462 |  | -0.04 | -0.83;0.76 | 0.929 |
|  |  |  | 60-69 | 0.90 | -0.02;1.82 | 0.054 |  | **1.17** | **0.4;1.93** | **0.003** |
|  |  | 50-59 | 60-69 | 0.53 | -0.32;1.38 | 0.222 |  | **1.20** | **0.46;1.94** | **0.002** |
|  | >60 min | 20-29 | 30-39 | -0.08 | -1.01;0.85 | 0.867 |  | 0.11 | -0.47;0.7 | 0.707 |
|  |  |  | 40-49 | -0.36 | -1.26;0.54 | 0.434 |  | -0.30 | -0.86;0.26 | 0.291 |
|  |  |  | 50-59 | -0.10 | -0.98;0.77 | 0.818 |  | -0.18 | -0.73;0.37 | 0.530 |
|  |  |  | 60-69 | 0.06 | -0.79;0.91 | 0.891 |  | -0.09 | -0.62;0.45 | 0.754 |
|  |  | 30-39 | 40-49 | -0.28 | -0.94;0.38 | 0.409 |  | -0.41 | -0.87;0.05 | 0.079 |
|  |  |  | 50-59 | -0.02 | -0.65;0.61 | 0.943 |  | -0.29 | -0.74;0.16 | 0.209 |
|  |  |  | 60-69 | 0.14 | -0.45;0.73 | 0.644 |  | -0.20 | -0.63;0.24 | 0.372 |
|  |  | 40-49 | 50-59 | 0.26 | -0.33;0.84 | 0.389 |  | 0.13 | -0.29;0.54 | 0.554 |
|  |  |  | 60-69 | 0.42 | -0.12;0.96 | 0.128 |  | 0.21 | -0.18;0.61 | 0.291 |
|  |  | 50-59 | 60-69 | 0.16 | -0.34;0.66 | 0.524 |  | 0.09 | -0.3;0.48 | 0.649 |
| CI=confidence interval | |  |  |  |  |  |  |  |  |  |

| Supplementary table 3. Associations between different bout lengths of stationary behavior and CVD risk factors. | | | | | | | | | | | | | | | |  |  |  |  |  |  |  |  |  |
| --- | --- | --- | --- | --- | --- | --- | --- | --- | --- | --- | --- | --- | --- | --- | --- | --- | --- | --- | --- | --- | --- | --- | --- | --- |
|  |  | CVD risk score (FRS) | | |  | HDL-cholesterol | | |  | LDL-cholesterol | | |  | Total-cholesterol | | |  | Triglyceride | | |  | Waist circumference | | |
|  | bout length | B* | 95% CI | p-value |  | B* | 95% CI | p-value |  | B* | 95% CI | p-value |  | B* | 95% CI | p-value |  | B* | 95% CI | p-value |  | B* | 95% CI | p-value |
| Lying | <3 min | 9.95 | 0.64;13.49 | **<0.001** |  | -0.67 | -1.10;-0.25 | **0.002** |  | 0.02 | -0.18;0.22 | 0.019 |  | -0.05 | -0.23;0.14 | 0.634 |  | 0.88 | 0.60;1.17 | **<0.001** |  | 0.07 | 0.06;0.08 | **<0.001** |
|  | <5 min | 16.78 | 11.12;22.43 | **<0.001** |  | -1.35 | -2.03;-0.68 | **<0.001** |  | 0.09 | -0.23;0.42 | 0.568 |  | -0.06 | -0.36;0.24 | 0.716 |  | 1.51 | 1.06;1.96 | **<0.001** |  | 0.12 | 0.09;0.14 | **<0.001** |
|  | <10 min | 29.69 | 19.68;39.70 | **<0.001** |  | -2.40 | -3.58;-1.21 | **<0.001** |  | 0.17 | -0.40;0.73 | 0.567 |  | -0.06 | -0.58;0.47 | 0.831 |  | 2.91 | 2.12;3.70 | **<0.001** |  | 0.21 | 0.17;0.24 | **<0.001** |
|  | <20 min | 52.13 | 35.77;68.48 | **<0.001** |  | -4.13 | -6.05;-2.21 | **<0.001** |  | 0.51 | 0.41;1.43 | 0.479 |  | 0.21 | -0.65;1.06 | 0.636 |  | 5.14 | 3.86;6.42 | **<0.001** |  | 0.32 | 0.26;0.38 | **<0.001** |
|  | <30 min | 69.51 | 48.30;90.72 | **<0.001** |  | -5.95 | -8.41;-3.48 | **<0.001** |  | 0.43 | -0.75;1.61 | 0.473 |  | 0.09 | -1,00;1.19 | 0.868 |  | 6.73 | 5.09;8.37 | **<0.001** |  | 0.38 | 0.30;0.46 | **<0.001** |
|  | <60 min | 105.69 | 76.75;134.62 | **<0.001** |  | -7.93 | -11.28;-4.59 | **<0.001** |  | 1.01 | -0.59;2.61 | 0.216 |  | 0.65 | 0.84;2.13 | 0.392 |  | 9.18 | 6.96;11.41 | **<0.001** |  | 0.44 | 0.33;0.54 | **<0.001** |
|  |  |  |  |  |  |  |  |  |  |  |  |  |  |  |  |  |  |  |  |  |  |  |  |  |
|  | ≥60 min | 42.65 | 29.41;55.88 | **<0.001** |  | -2.44 | -4.02;-0.88 | **0.002** |  | 0.54 | -0.21;1.29 | 0.155 |  | 0.50 | 0.20;1.19 | 0.161 |  | 2.24 | 1.19;3.28 | **<0.001** |  | 0.05 | 0.00;010 | **0.043** |
|  | ≥30 min | 78.82 | 57.66;99.99 | **<0.001** |  | -4.43 | -6.93;-1.94 | **<0.001** |  | 1.12 | -0.07;2.31 | 0.065 |  | 1.05 | -0.05;2.15 | 0.062 |  | 4.69 | 3.03;6.36 | **<0.001** |  | 0.11 | 0.03;0.19 | **0.007** |
|  | ≥20 min | 96.21 | 70.97;121.44 | **<0.001** |  | -6.25 | -9.21;-3.29 | **<0.001** |  | 1.05 | -0.37;2.46 | 0.147 |  | 0.94 | -0.37;2.25 | 0.160 |  | 6.28 | 4.30;8.25 | **<0.001** |  | 0.17 | 0.08;0.26 | **<0.001** |
|  | ≥10 min | 118.64 | 88.79;148.50 | **<0.001** |  | -7.98 | -11.47;-4.50 | **<0.001** |  | 1.39 | -0.28;3.06 | 0.103 |  | 1.20 | -0.34;2.74 | 0.127 |  | 8.51 | 6.19;10.83 | **<0.001** |  | 0.28 | 0.17;0.40 | **<0.001** |
|  | ≥5 min | 131.56 | 99.03;164.08 | **<0.001** |  | -9.03 | -12.83;-5.22 | **<0.001** |  | 1.46 | -0.36;3.28 | 0.116 |  | 1.20 | -0.49;2.88 | 0.163 |  | 9.91 | 7.38;12.44 | **<0.001** |  | 0.37 | 0.25;0.50 | **<0.001** |
|  | ≥3 min | 138.39 | 104.80;171.98 | **<0.001** |  | -9.70 | -13.63;-5.78 | **<0.001** |  | 1.53 | -0.35;3.41 | 0.109 |  | 1.19 | -0.55;2.93 | 0.180 |  | 10.54 | 7.93;13.15 | **<0.001** |  | 0,42 | 0.30;0.55 | **<0.001** |
|  | ≥1 min | 145.89 | 111.18; 180.60 | **<0.001** |  | -10.27 | -14.33; -6.21 | **<0.001** |  | 1.55 | -0.39; 3.49 | 0.118 |  | 1.15 | -0.65; 2.95 | 0.210 |  | 11.23 | 8.54;13.93 | **<0.001** |  | 0.47 | 0.35; 0.60 | **<0.001** |
| Reclining | <3 min | 12.21 | -0.27,2.03 | **0.007** |  | -1.78 | -2.80;-0.75 | **0.001** |  | 0.21 | -0.28;0.70 | 0.404 |  | -0.32 | -0.77;0.14 | 0.169 |  | 1.28 | 0.60;1.97 | **<0.001** |  | 0.18 | -0.60;1.09 | **<0.001** |
|  | <5 min | 20.39 | 6.18,34.59 | **0.005** |  | -3.77 | -5.40;-2.13 | **<0.001** |  | 0.38 | -0.40;1.17 | 0.338 |  | -0.59 | -1.31;0.14 | 0.112 |  | 2.17 | 1.08;3.27 | **<0.001** |  | 0.30 | 0.25;2.57 | **<0.001** |
|  | <10 min | 42.32 | 18.14;66.40 | **0.001** |  | -8.19 | -10.95;-5.43 | **<0.001** |  | 1.03 | -0.30;2.35 | 0.129 |  | -0.63 | -1.86;0.59 | 0.312 |  | 5.03 | 3.18;6.87 | **<0.001** |  | 0.54 | 0.46;0.63 | **<0.001** |
|  | <20 min | 97.58 | 61.46;133.70 | **<0.001** |  | -15.70 | -19.86;-11.53 | **<0.001** |  | 1.48 | -0.53;3.48 | 0.149 |  | -0.70 | -2.55;1.16 | 0.460 |  | 9.75 | 6.97;12.54 | **<0.001** |  | 0.96 | 0.83;1.09 | **<0.001** |
|  | <30 min | 145.43 | 101.90;188.95 | **<0.001** |  | -21.30 | -26.32;-16.28 | **<0.001** |  | 1.58 | -0.85;4.01 | 0.202 |  | -0.99 | -3.23;1.25 | 0.388 |  | 12.88 | 9.51;16.25 | **<0.001** |  | 1.28 | 1,12;1.43 | **<0.001** |
|  | <60 min | 223.48 | 168.87;278.09 | **<0.001** |  | -31.12 | -37.41;-24.85 | **<0.001** |  | 2.38 | -0.67;5.42 | 0.126 |  | 0.93 | -3.75;1.88 | 0.516 |  | 18.18 | 13.96;22.40 | **<0.001** |  | 1.76 | 1.57;1.96 | **<0.001** |
|  |  |  |  |  |  |  |  |  |  |  |  |  |  |  |  |  |  |  |  |  |  |  |  |  |
|  | ≥60 min | 34.34 | 10.14;58.54 | **0.005** |  | -3.87 | -6.65;-1.10 | **0.006** |  | 0.44 | -0.89;1.76 | 0.518 |  | 0.53 | -0.70;1.75 | 0.402 |  | 3.43 | 1.58;5.29 | **<0.001** |  | 0.38 | 0.29;0.47 | **<0.001** |
|  | ≥30 min | 112.39 | 72.20;152.58 | **<0.001** |  | -13.70 | -18.31;-9.10 | **<0.001** |  | 1.23 | -0.98;3.44 | 0.274 |  | 0.58 | -1.46;2.62 | 0.577 |  | 8.73 | 5.65;11.81 | **<0.001** |  | 0.86 | 0.72;1.01 | **<0.001** |
|  | ≥20 min | 160.24 | 112.88;207.61 | **<0.001** |  | -19.31 | -24.75;-13.86 | **<0.001** |  | 1.34 | -1.28;3.96 | 0.317 |  | 0.29 | -2.13;2.71 | 0.813 |  | 11.85 | 8.21;15.50 | **<0.001** |  | 1.18 | 1,01;1,36 | **<0.001** |
|  | ≥10 min | 215.50 | 159.34;271.66 | **<0.001** |  | -26.81 | -33.28;-20.35 | **<0.001** |  | 1.79 | -1.33;4.90 | 0.261 |  | 0.22 | -2.66;3.11 | 0.879 |  | 16.58 | 12.25;20.91 | **<0.001** |  | 1.60 | 1,40;1.80 | **<0.001** |
|  | ≥5 min | 237,43 | 177.20;297.66 | **<0.001** |  | -31.24 | 38.17;-24.30 | **<0.001** |  | 2.43 | -0.92;5.78 | 0.155 |  | 0.18 | -2.29;3.28 | 0.909 |  | 19.44 | 14.79;24.08 | **<0.001** |  | 1.84 | 1,63;2.06 | **<0.001** |
|  | ≥3 min | 245,61 | 183.68;307.54 | **<0.001** |  | -33.23 | -40.36;-26.10 | **<0.001** |  | 2.60 | -0.84;6.05 | 0.139 |  | -0.09 | -3.28;3.10 | 0.956 |  | 20.33 | 15.55;25.11 | **<0.001** |  | 1.96 | 1.74;2.18 | **<0.001** |
|  | ≥1 min | 254.42 | 191.19; 317.66 | **<0.001** |  | -34.75 | -42.03; -27.47 | **<0.001** |  | 2.77 | -0.76; 6.29 | 0.124 |  | -0.36 | -3.62; 2.90 | 0.830 |  | 21.37 | 16.49;26.24 | **<0.001** |  | 2.10 | 1.88; 2.33 | **<0.001** |
| Sitting | <3 min | -44.66 | -56.38;-32.95 | **<0.001** |  | 3.78 | 2.43;5.13 | **<0.001** |  | -0.70 | -1.35;-0.06 | **0.033** |  | -0.84 | -1.44;-0.24 | **0.006** |  | -4.27 | -5.17;-3.38 | **<0.001** |  | -0.31 | -0.35;-0.27 | **<0.001** |
|  | <5 min | -70.83 | -88.10;-53.56 | **<0.001** |  | 5.73 | 3.73;7.72 | **<0.001** |  | -1.23 | -2.18;-0.27 | **0.012** |  | -1.28 | -2.17;-0.40 | **0.004** |  | -6.23 | -7.55;-4.91 | **<0.001** |  | -0.45 | -0.52;-0.39 | **<0.001** |
|  | <10 min | -108.38 | -133.97;-82.79 | **<0.001** |  | 7.31 | 4.35;10.28 | **<0.001** |  | -1.93 | -3.35;-0.51 | **0.008** |  | -1.88 | -3.20;-0.57 | **0.005** |  | -8.38 | -10.36;-6.42 | **<0.001** |  | -0.63 | -0.72;-0.54 | **<0.001** |
|  | <20 min | -143.87 | -177.86;-109.88 | **<0.001** |  | 8.55 | 4.60;12.50 | **<0.001** |  | -2.77 | -4.66;-0.89 | **0.004** |  | -2.40 | -4.15;-0.66 | **0.007** |  | -9.10 | -11.73;-6.47 | **<0.001** |  | -0.69 | -0.81;-0.57 | **<0.001** |
|  | <30 min | -155.17 | -193.57;-116.77 | **<0.001** |  | 8.58 | 4.13;13.03 | **<0.001** |  | -3.14 | -5.26;-1.01 | **0.004** |  | -2.63 | -4.60;-0.67 | **0.009** |  | -9.12 | -12.09;-6.16 | **<0.001** |  | -0.66 | -0,80,-0.52 | **<0.001** |
|  | <60 min | -166.21 | -209.60;-122.83 | **<0.001** |  | 7.71 | 2.69;12.73 | **0.003** |  | -3.46 | 5.86;-1.07 | **0.005** |  | -2.78 | -5.00;-0.57 | **0.014** |  | -8.16 | -11.51;-4.81 | **<0.001** |  | -0.58 | -0.74;-0.43 | **<0.001** |
|  |  |  |  |  |  |  |  |  |  |  |  |  |  |  |  |  |  |  |  |  |  |  |  |  |
|  | ≥60 min | 2.68 | -3.82;9.19 | 0.419 |  | 0.21 | -0.45;0.97 | 0.592 |  | 0.14 | 0.23;0.51 | 0.453 |  | 0.070 | -0.27;0.40 | 0.706 |  | 0.07 | -0.44;0.58 | 0.792 |  | 0.03 | 0.01;0.06 | **0.011** |
|  | ≥30 min | -8.36 | -22.78;6.07 | 0.256 |  | -0.66 | -2.30;0.99 | 0.434 |  | -0.19 | -0.97;0.60 | 0.641 |  | -0.08 | -0.81;0.64 | 0.820 |  | 1.03 | -0.07;2.13 | 0.065 |  | 0.11 | 0.06;0.17 | **<0.001** |
|  | ≥20 min | -19.66 | -40.01;0.69 | 0.058 |  | -0.63 | -2.95;1.70 | 0.597 |  | -0.55 | -1.66;0.56 | 0.331 |  | -0.31 | -1.34;0.71 | 0.548 |  | 1.01 | -0.54;2.56 | 0.203 |  | 0.14 | 0.06;0.22 | **<0.001** |
|  | ≥10 min | -55.15 | -85.43;-24.87 | **<0.001** |  | 0.61 | -2.85;4.08 | 0.729 |  | -1.39 | -3.05;0.26 | 0.100 |  | -0.83 | -2.36;0.70 | 0.285 |  | 0.3 | -2.02;2.62 | 0.799 |  | 0.08 | -0.03;0.19 | 0.169 |
|  | ≥5 min | -92.69 | -130.02;-55.37 | **<0.001** |  | 2.20 | -2.09;6.48 | 0.315 |  | -2.10 | -4.14;-0.05 | **0.045** |  | -1.44 | -3.33;0.46 | 0,137 |  | -1.86 | -4.72;1.01 | 0.204 |  | -0.10 | -0.23,0.04 | 0.173 |
|  | ≥3 min | -118.86 | -159.53;-78.20 | **<0.001** |  | 4.14 | -0.54;8.82 | 0.083 |  | -2.62 | -4.85;-0.38 | **0.022** |  | -1.88 | -3.94;0.19 | 0,075 |  | -3.82 | -6.95;-0.69 | 0.017 |  | -0.24 | -0.39;-0.10 | **0.001** |
|  | ≥1 min | -153-23 | -197.66; -108..79 | **<0.001** |  | 6.96 | 1.83; 12.09 | **0.008** |  | -3.10 | -5.55; -0.65 | **0.013** |  | -2.48 | -4.74; -0.21 | **0.032** |  | -7.01 | -10.43;-3.58 | **<0.001** |  | -0.48 | -0.64; -0.32 | **<0.001** |
| Standing | <3 min | -62.68 | -76.83; -48.53 | **<0.001** |  | 8.46 | 6.85; 10.07 | **<0.001** |  | -0.79 | -1.57; -0.01 | **0.048** |  | -0.39 | -1.11; 0.33 | 0.286 |  | -6.36 | -7.43;-5.29 | **<0.001** |  | -0.54 | -0.59; -0.49 | **<0.001** |
|  | <5 min | -90.70 | -111.06; -70.35 | **<0.001** |  | 12.08 | 9.77; 14.38 | **<0.001** |  | -1.13 | -2.25; -0.02 | **0.047** |  | -0.57 | -1.60; 0.47 | 0.282 |  | -8.98 | -10.51;-7.45 | **<0.001** |  | -0.77 | -0.84; -0.70 | **<0.001** |
|  | <10 min | -121.63 | -150.03; -93.23 | **<0.001** |  | 16.82 | 13.64; 20.00 | **<0.001** |  | -1.58 | -3.12; -0.03 | **0.045** |  | -0.78 | -2.21; 0.65 | 0.286 |  | -12.17 | -14.29;-10.05 | **<0.001** |  | -1.04 | -1.13; -0.94 | **<0.001** |
|  | <20 min | -141.94 | -176.26; -107.62 | **<0.001** |  | 19.71 | 15.87; 23.54 | **<0.001** |  | -1.83 | -3.69; 0.03 | 0.053 |  | -0.93 | -2.65; 0.79 | 0.290 |  | -13.98 | -16.53;-11.42 | **<0.001** |  | -1.19 | -1.30; -1.07 | **<0.001** |
|  | <30 min | -147.77 | -184.21; -111.34 | **<0.001** |  | 20.44 | 16.38;24.51 | **<0.001** |  | -1.97 | -3.93; 0.01 | **0.050** |  | -1.08 | -2.90; 0.74 | 0.246 |  | -14.43 | -17.15;-11.72 | **<0.001** |  | -1.23 | -1.35; -1.10 | **<0.001** |
|  | <60 min | -156.06 | -194.58;-117.54 | **<0.001** |  | 21.26 | 16.96;25.55 | **<0.001** |  | -1.97 | -4.05;0.11 | 0.064 |  | -1.08 | -3.00;0.85 | 0.274 |  | -14.71 | -17.58;-11.84 | **<0.001** |  | -1.26 | -1.39;1.31 | **<0.001** |
|  |  |  |  |  |  |  |  |  |  |  |  |  |  |  |  |  |  |  |  |  |  |  |  |  |
|  | ≥60 min | 2.46 | -5.22;0.31 | 0.082 |  | 0.33 | 0.01;0.65 | **0.045** |  | 0.04 | 0.11;0.20 | 0.586 |  | 0.04 | -0.11;0.18 | 0.628 |  | -0.14 | -0.36;0.07 | 0.195 |  | -0.01 | -0.02;-0.00 | **0.030** |
|  | ≥30 min | -10.74 | -17.73; -3.74 | **0.003** |  | 1.14 | 0.35; 1.94 | 0.352 |  | 0.04 | -0.34; 0.42 | 0.838 |  | 0.04 | -0.31; 0.39 | 0.829 |  | -0.42 | --0.95;0.11 | 0.124 |  | -0.05 | -0.07; -0.02 | **<0.001** |
|  | ≥20 min | -16.57 | -26.86; -6.28 | **0.002** |  | 1.88 | 0.73; 3.02 | **0.001** |  | -0.10 | -0.64; 0.45 | 0.734 |  | -0.11 | -0.62; 0.40 | 0.667 |  | -0.87 | -1.64;-0.11 | **0.026** |  | -0.09 | -0.12; -0.05 | **<0.001** |
|  | ≥10 min | -36.88 | -54.22; -19.54 | **<0.001** |  | 4.77 | 2.83; 6.70 | **<0.001** |  | -0.35 | -1.28; 0.58 | 0.459 |  | -0.26 | -1.12; 0.60 | 0.549 |  | -2.68 | -3.97;-1.38 | **<0.001** |  | -0.24 | -0.30; -0.18 | **<0.001** |
|  | ≥5 min | -67.81 | -92.50; -43.12 | **<0.001** |  | 9.51 | 6.77; 12.25 | **<0.001** |  | -0.79 | -2.11; 0.53 | 0.238 |  | -0.47 | -1.69; 0.75 | 0.447 |  | -5.87 | -7.71;-4.04 | **<0.001** |  | -0.50 | -0.59; -0.42 | **<0.001** |
|  | ≥3 min | -95.83 | -125.49; -66.18 | **<0.001** |  | 13.12 | 9.83; 16.42 | **<0.001** |  | -1.14 | -2.73; 0.45 | 0.160 |  | -0.65 | -2.12; 0.82 | 0.387 |  | -8.49 | -10.70;-6.29 | **<0.001** |  | -0.73 | -0.83; '0.63 | **<0.001** |
|  | ≥1 min | -142.30 | -179.21; -105.38 | **<0.001** |  | 19.63 | 15.52; 23.74 | **<0.001** |  | -1.69 | -3.68; 0.29 | 0.095 |  | -0.90 | -2.74; 0.94 | 0.336 |  | -13.27 | -16.02;-10.53 | **<0.001** |  | -1.14 | -1.27; '1.02 | **<0.001** |
| *non-standardized regression coefficient | | | | |  |  |  |  |  |  |  |  |  |  |  |  |  |  |  |  |  |  |  |  |
| CVD=cardiovascular disease, FRS=Framingham risk score, HDL=high-density lipoprotein, LDL=low-density lipoprotein, CI=confidence interval | | | | | | | | | | | | | | | | | | | |  |  |  |  |  |
